# Rapid identification of genes controlling virulence and immunity in malaria parasites

**DOI:** 10.1371/journal.ppat.1006447

**Published:** 2017-07-12

**Authors:** Hussein M. Abkallo, Axel Martinelli, Megumi Inoue, Abhinay Ramaprasad, Phonepadith Xangsayarath, Jesse Gitaka, Jianxia Tang, Kazuhide Yahata, Augustin Zoungrana, Hayato Mitaka, Arita Acharjee, Partha P. Datta, Paul Hunt, Richard Carter, Osamu Kaneko, Ville Mustonen, Christopher J. R. Illingworth, Arnab Pain, Richard Culleton

**Affiliations:** 1 Malaria Unit, Department of Pathology, Institute of Tropical Medicine, Nagasaki University, Nagasaki, Japan; 2 Institute of Immunology and Infection Research, School of Biological Sciences, University of Edinburgh, Edinburgh, United Kingdom; 3 Global Station for Zoonosis Control, Global Institution for Collaborative Research and Education (GI-CoRE), Hokkaido University, Sapporo, Japan; 4 Biological and Environmental Sciences and Engineering (BESE) Division, King Abdullah University of Science and Technology (KAUST), Thuwal, Saudi Arabia; 5 Graduate School of Biomedical Sciences, Nagasaki University, Nagasaki, Japan; 6 Department of Protozooolgy, Institute of Tropical Medicine, Nagasaki University, Nagasaki, Japan; 7 Centre for Malaria Elimination, School of Medicine, Mount Kenya University, Thika, Kenya; 8 Key Laboratory of National Health and Family Planning Commission on Parasitic Disease Control and Prevention, Jiangsu Provincial Key Laboratory on Parasite and Vector Control Technology, Jiangsu Institute of Parasitic Diseases, Jiangsu, China; 9 Indian Institute of Science Education and Research Kolkata, Mohanpur - 741 246, West Bengal, India; 10 Wellcome Trust Sanger Institute, Hinxton, United Kingdom; 11 Department of Genetics, University of Cambridge, Cambridge, United Kingdom; 12 Department of Applied Mathematics and Theoretical Physics, University of Cambridge, Cambridge, United Kingdom; Texas Biomedical Research Institute, UNITED STATES

## Abstract

Identifying the genetic determinants of phenotypes that impact disease severity is of fundamental importance for the design of new interventions against malaria. Here we present a rapid genome-wide approach capable of identifying multiple genetic drivers of medically relevant phenotypes within malaria parasites via a single experiment at single gene or allele resolution. In a proof of principle study, we found that a previously undescribed single nucleotide polymorphism in the binding domain of the erythrocyte binding like protein (EBL) conferred a dramatic change in red blood cell invasion in mutant rodent malaria parasites *Plasmodium yoelii*. In the same experiment, we implicated merozoite surface protein 1 (MSP1) and other polymorphic proteins, as the major targets of strain-specific immunity. Using allelic replacement, we provide functional validation of the substitution in the EBL gene controlling the growth rate in the blood stages of the parasites.

## Author summary

Developing a greater understanding of malaria genetics is a key step in combating the threat posed by the disease. Here we use a novel approach to study two important properties of the parasite; the rate at which parasites grow within a single host, and the means by which parasites are affected by the host immune system. Two malaria strains with different biological properties were crossed in mosquitoes to produce a hybrid population, which was then grown in naïve and vaccinated mice. Parasites with genes conveying increased growth or immune evasion are favoured under natural selection, leaving a signature on the genetic composition of the cross population. We describe a novel mathematical approach to interpret this signature, identifying selected genes within the parasite population. We discover new genetic variants conveying increased within-host growth and resistance to host immunity in a mouse malaria strain. Experimental validation highlights the ability of this rapid experimental process for generating insights into malaria biology.

## Introduction

Malaria parasite strains are genotypically polymorphic, leading to a diversity of phenotypic characteristics that impact on disease severity. Discovering the genetic basis for such phenotypic traits can inform the design of new drugs and vaccines. Both association mapping and linkage analyses approaches have been adopted to understand the genetic mechanisms behind various phenotypes of malaria parasites [1–5] and with the application of whole genome sequencing (WGS), the resolution of these methodologies has been dramatically improved, allowing the discovery of selective sweeps as they arise in the field [6]. However, both approaches suffer from drawbacks when working with malaria parasites: linkage mapping requires the cloning of individual recombinant offspring, a process that is both laborious and time-consuming, and association studies require the collection of a large number of individual parasites (usually in the thousands) from diverse geographical origins and over periods of several months or years to produce enough resolution for the detection of selective sweeps.

Linkage Group Selection (LGS), like linkage mapping, relies on the generation of genetic crosses, but bypasses the need for extracting and phenotyping individual recombinant clones. Instead, it relies on quantitative molecular markers to measure allele frequencies in the recombinant progeny and identify loci under selection [7, 8]. This approach bears similarity to Bulked Segregant Analysis (BSA) [9], a technique developed to study disease resistance in plants. In BSA, individuals from a population are segregated based upon their phenotype (e.g. disease resistance), following which the frequencies of genetic markers in each population are analysed, identifying loci at which different alleles are found for the differently phenotypes populations. Segregating individuals by phenotype, while relatively straight forward for large organisms such as plants, is not feasible for unicellular pathogens such as malaria parasites. Instead, in LGS, the segregating population is grown both in the presence or absence of a selection pressure (e.g. drug treatment, immune pressure, etc.). Selection removes susceptible individuals in the selected “pool”, while leaving both susceptible and resistant individuals in the unselected “pool”. In its original implementation, LGS was successfully applied in studying strain-specific immunity (SSI) [10, 11], drug resistance [7, 12] and growth rate [8] in malaria and SSI in *Eimeria tenella* [13]. LGS is essentially identical to the extreme QTL approach (xQTL) that was independently developed by yeast researchers based on BSA [14].

In both the original implementations of BSA and LGS a limiting factor is the availability of molecular markers differentiating the two populations. One step in increasing the number of molecular markers was through the use of array hybridisation that allowed the identification of thousands of SNPs as molecular markers in *Arabidopsis thaliana* [15]. BSA (still using pre-selected pools) was also combined with tiling microarray hybridisation and used probe intensities to detect a gene underlying xylose utilisation in yeast [16]. The xQTL method increased the power and rapidity of the approach by making use of available yeast microarray data as well as Next Generation Sequencing (NGS) of DNA hybridised to microarray probes to identify a large number of markers across the genome, this time comparing selected and unselected populations, rather then generating pools based on phenotype [14, 17]. In the absence of microarray databases, an alternative approach was to use NGS short reads to identify genome-wide SNPs between two parents and then use these SNPs as molecular markers to identify target genes in the selected progeny population compared against the unselected population, as done to study chloroquine resistance in malaria [18].

**Fig 1:**
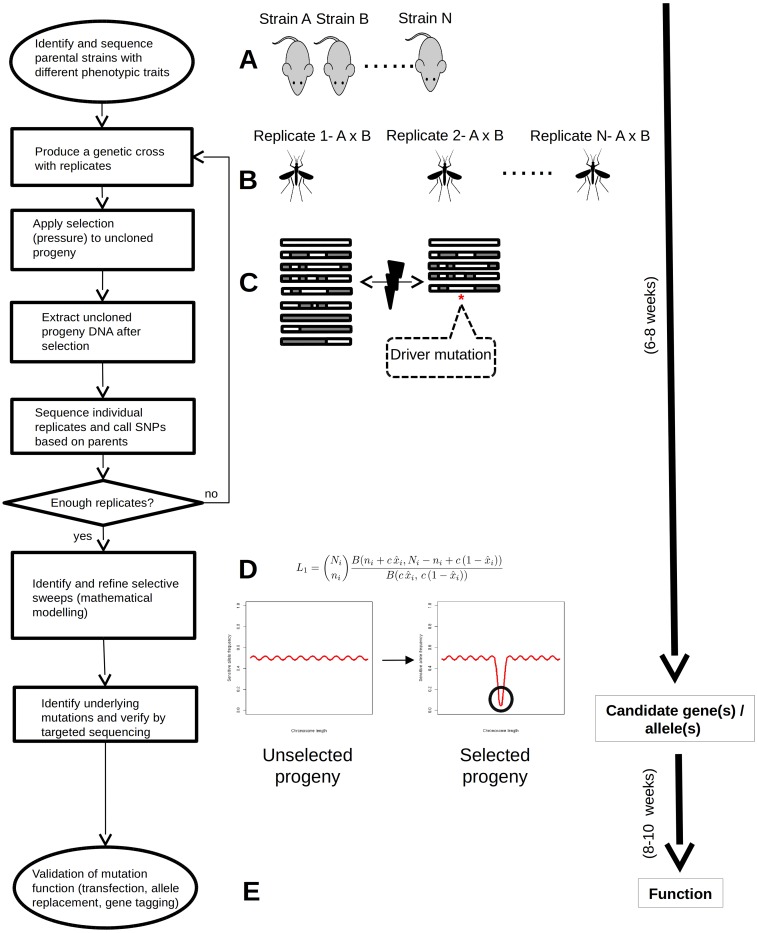
Schematic representation of the multi-crossing LGS approach. The process starts with the identification of distinct selectable phenotypes in cloned strains of the pathogen population (in this case malaria parasites) and their sequencing, usually from the vertebrate blood stage. A genetic cross between two cloned strains is subsequently produced, in this case inside the mosquito vector. The cross progeny is then grown with and without selection pressure(s). Selection pressure will remove those progeny individuals carrying allele(s) associated with sensitivity to the selection pressure(s), while allowing progeny individuals with the resistant allele(s) to survive. DNA is then extracted from the whole, uncloned progeny for sequencing. SNPs distinguishing both parents are used to measure allele frequencies in the selected and unselected progenies. A mathematical model is then applied to identify and define loci under selection. Regions in these loci are then analyzed in detail to identify potential target polymorphisms underlying the phenotype(s) under investigation. Targeted capillary sequencing can be employed to verify or further characterize polymorphisms. Finally, where applicable, allele replacement experiments can be carried out to confirm the effect of target polymorphisms.

In this study, we apply an improved LGS approach for the identification of genes controlling two independent and naturally occurring phenotypic differences between two strains of the rodent malaria parasite *Plasmodium yoelii*; growth rate, and strain-specific immunity. A mathematical model, built upon methodological improvements in the analysis of genetically crossed populations [19, 20], was developed to analyze the data. This modified LGS approach relies on the generation and selection of at least two independent crosses between the strains. The progeny from both crosses pre- and post-selection are then subjected to high-throughput WGS, and SNP marker movement analyzed using best fitting modeling (Fig 1).

Our novel statistical framework both accounts for the influence of clonal growth in the cross population, and allows for a locally variable recombination rate in the parasite population, unlike previous analyses applied to comparable data [21]. Applying this framework to crosses between two strains of *P. yoelii* that induce SSI, and which differ in their growth rates, we were able to identify three genomic regions and alleles controlling both phenotypes, demonstrating that the approach can be used to analyze multiple complex phenotypes concomitantly with high genomic resolution within a short space of time.

## Results

### Characterization of strain specific immunity and growth rate phenotypic differences between CU and 17X1.1pp

**Fig 2:**
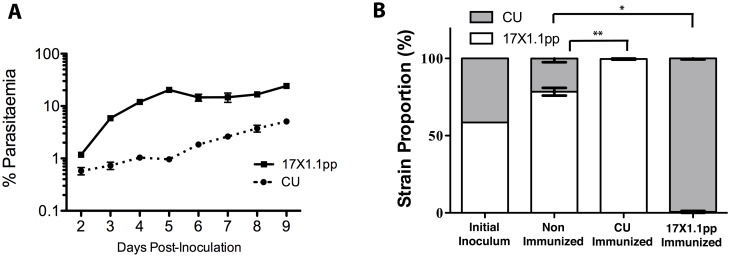
Pure strain growth rates. **(A)** Growth rate of *Plasmodium yoelii* strains 17X1.1pp and CU in CBA mice inoculated with 1 × 10^6^ iRBCs on Day 0. Error bars indicate the standard error of the mean for three mice per group. **(B)** The relative proportions of CU and 17X1.1pp were measured by Q-RT-PCR targeting the polymorphic *msp1* locus at Day 4 post-inoculation with a mixed inoculum containing approximately equal proportions of both strains in naïve mice and mice immunized with one of the two strains. Error bars show the standard error of the mean of five mice per group. *p<0.05, Wilcoxon rank sum test, W = 25, p = 0.0119, n = 5; ** p<0.01, Wilcoxon rank sum test, W = 25, p = 0.0075, n = 5

The difference in blood-stage parasite growth rate between the two clones was followed *in vivo* for nine days in CBA mice. A likelihood ratio test using general linear mixed models indicated a more pronounced growth rate for 17X1.1pp compared to CU clone by time interaction term, L = 88.60, df = 21, p<0.0001, Fig 2A). To verify that the two malaria clones could also be used to generate protective SSI, groups of mice were immunized with 17X1.1pp, CU or mock immunized, prior to challenge with a mixture of the two clones (S1 Fig). The relative proportions of the two clones were measured on day four of the infection by real time quantitative PCR (Q-RT-PCR) targeting the polymorphic *msp1* locus [22]. A strong, statistically significant SSI was induced by both parasite strains in CBA mice (Fig 2B).

### Identification of high-confidence SNPs

Two kinds of selection pressure were applied in this study: growth rate driven selection and SSI. Two independent genetic crosses between 17X1.1pp and CU were produced, and both these crosses were subjected to immune selection (in which the progeny were grown in mice made immune to either of the two parental clones), and grown in non-immune mice. Progeny were harvested from mice four days after challenge, at which point strain-specific immune selection in the immunized mice, and selection of faster growing parasites in the non-immune mice had occurred. Using deep sequencing by Illumina technology, a total of 29,053 high confidence genome-wide SNPs that distinguish the parental strains were produced by read mapping with custom-made Python scripts. SNP frequencies from these loci from each population were filtered using a likelihood ratio test to remove sites where alleles had been erroneously mapped to the wrong genome location.

### Identification of clonality within the data

A hidden Markov model was applied to the data to identify allele frequency changes (Table 1) that were likely to have arisen from the clonal growth of individuals within the cross population or possible incorrect assembly of the reference genome, as described in the Materials and Methods section and in more detail in the supplementary mathematical methods (S1 Appendix). In a genetic cross population, an especially high fitness clone generated by random recombination events can grow to substantial frequency, this being manifested as sudden jumps in allele frequency occurring at the recombination points in this individual [23]. Jumps of this type were primarily identified in the 17X-immunized population, where the increased virulence of the 17X strain had less of an effect in driving alleles to high frequency, and in the first replica experiment; the data in the first experiment seemed to have been more affected by clonal growth in the population. The consistency of identified jumps between treatment conditions reflects the common origin of the differently treated populations; the jump at the end of chromosome XIV inferred in both replicas may be artefactual.

**Table 1:** Sudden changes in allele frequency identified using a jump-diffusion model. Details are given for loci at which a sudden jump in frequency was inferred with probability at least 1%. The latter value is the inferred probability that the change in allele frequency at a given locus arose from a jump to a random position between 0 and 1, as opposed to arising from a small change to the frequency at the previous locus. Data are shown for the naïve and 17-X immunized experiments; no jumps of this significance were inferred for the CU-immunized experiment.

Condition	Replica	Chromosome	x_i_	p_i_
Naïve	1	IX	861097	0.011
Naïve	1	XIII	1792073	0.032
Naïve	1	XIV	2511246	0.494
Naïve	2	XIV	2511246	0.144
17X-immunised	1	I	632854	0.081
17X-immunised	1	II	41486	0.046
17X-immunised	1	II	275583	0.028
17X-immunised	1	II	414858	0.157
17X-immunised	1	III	126794	0.098
17X-immunised	1	III	351254	0.045
17X-immunised	1	IV	336683	0.456
17X-immunised	1	V	154909	0.637
17X-immunised	1	V	265353	0.706
17X-immunised	1	V	427343	0.245
17X-immunised	1	V	456836	0.086
17X-immunised	1	VI	776020	0.276
17X-immunised	1	VI	1020341	0.169
17X-immunised	1	VI	1034851	0.023
17X-immunised	1	VII	569214	0.256
17X-immunised	1	VIII	31296	0.166
17X-immunised	1	VIII	878879	0.209
17X-immunised	1	VIII	1387572	0.013
17X-immunised	1	IX	242610	0.180
17X-immunised	1	IX	867007	0.012
17X-immunised	1	X	602572	0.040
17X-immunised	1	XII	1090504	0.166
17X-immunised	1	XIII	1721212	0.017
17X-immunised	1	XIII	1784682	0.014
17X-immunised	1	XIII	1798425	0.019
17X-immunised	1	XIII	1818507	0.054
17X-immunised	1	XIV	582823	0.020
17X-immunised	1	XIV	1494755	0.090
17X-immunised	1	XIV	1692098	0.732
17X-immunised	1	XIV	2003325	0.260
17X-immunised	1	XIV	2511246	0.977
17X-immunised	2	II	41692	0.506
17X-immunised	2	VIII	27483	0.049
17X-immunised	2	XIV	2511246	0.231

### Identification of loci under selection

Based upon an analytical evolutionary model describing patterns of allele frequencies following selection, a maximum likelihood approach was used to define confidence intervals for the positions of alleles under selection in each of the genetic cross populations. In the absence of selection acting for a variant in a region of the genome, the allele frequencies in that region are expected to be locally constant. In common with a previous approach to identifying selected alleles [21], a search was therefore made for regions of the genome in which allele frequencies varied substantially according to their position in the genome. Next, wherever deviations of this form were consistently identified in both replica experiments a model of selection was applied to the data, inferring for each set of replica data the position in that region of the genome that was most likely to be under selection; this model was based upon expected changes in allele frequency under a constant local rate of recombination and is described further in the Methods section. Regions of the genome in which this inference of selection produced consistent results across replica datasets were then identified (Table 2). Of a total of 11 genomic regions suggesting evidence of non-neutrality, six showed sufficient evidence of consistent selection.

**Table 2:** Identification of candidate regions by non-neutrality score and SD model selected allele location. The non-neutrality score for region in replica *r* is denoted *S*_*r*_. The optimal driver location in the same region is given by $i_{r}^{*}$. Where a chromosome is divided into parts, by potential jump alleles, the resulting genomic regions are denoted by their chromosome number, a subscript indicating which part of the genome was under consideration. Identified candidate regions were defined as those at which selection was identified at positions within 200kb in both replicates, and are here highlighted in bold type.

Condition	Chromosome	Score S_1_	Score S_1_	Locus $i_{1}^{*}\;(\text{kb})$	Locus $i_{2}^{*}\;(\text{kb})$
**Naïve**	**XIII_1_ / XIII**	**0.85**	**1.45**	**1487.421**	**1426.749**
Naïve	VIII	0.12	0.10	1088.444	584.776
17X-immunised	XIII_2_ / XIII	7.96	9.30	1864.555	1327.063
**17X-immunised**	**XIII_1_ / XIII**	**1.16**	**9.30**	**1430.712**	**1327.063**
**17X-immunised**	**VIII_2_ / VIII**	**0.65**	**5.72**	**1289.719**	**1307.192**
17X-immunised	VIII_1_ / VIII	0.11	5.72	149.814	1307.192
**17X-immunised**	**VII_2_ / VII**	**0.25**	**1.35**	**617.952**	**786.643**
17X-immunised	VII_1_ / VII	0.15	1.35	442.278	786.643
**17X-immunised**	**IV_1_ / IV**	**0.10**	**1.25**	**304.778**	**173.740**
**CU-immunised**	**VIII**	**0.62**	**0.18**	**1327.639**	**1287.515**
CU-immunised	XIII	0.14	0.34	1602.238	1063.913

For each of these regions of the genome, a more sophisticated evolutionary model, accounting for variation in the local recombination rate, was then applied to the data, refining the position of the putatively selected allele. At this point, a putative selected allele in chromosome IV was removed from consideration, leaving five cases of potential alleles under selection in three regions of the genomes; confidence intervals for the positions of the selected loci are given in Table 3. Optimal positions of variant loci derived from each replicate are detailed in S1 Table; results of the variable recombination rate model are shown in S2 Table, with inferred recombination rates in S3 Table.

**Table 3:** Confidence intervals for driver locations as determined by mathematical modeling.

Condition	Selected Allele	Allele location	Combined interval (kb)	Conservative interval (kb)
Naïve	CU	XIII:1513.899	1513.899-1528.275	1436.717-1528.275
17X1.1pp-selected	17X1.1pp	VII:733.215	729.368-735.525	725.528-813.866
17X1.1pp-selected	17X1.1pp	VIII:1311.291	1306.083-1316.428	1229.582-1363.920
17X1.1pp-selected	CU	XIII:1480.699	1480.699-1489.964	1480.699-1528.275
CU-selected	CU	VIII:1294.402	1286.595-1302.526	1285.184-1332.198

**Fig 3:**
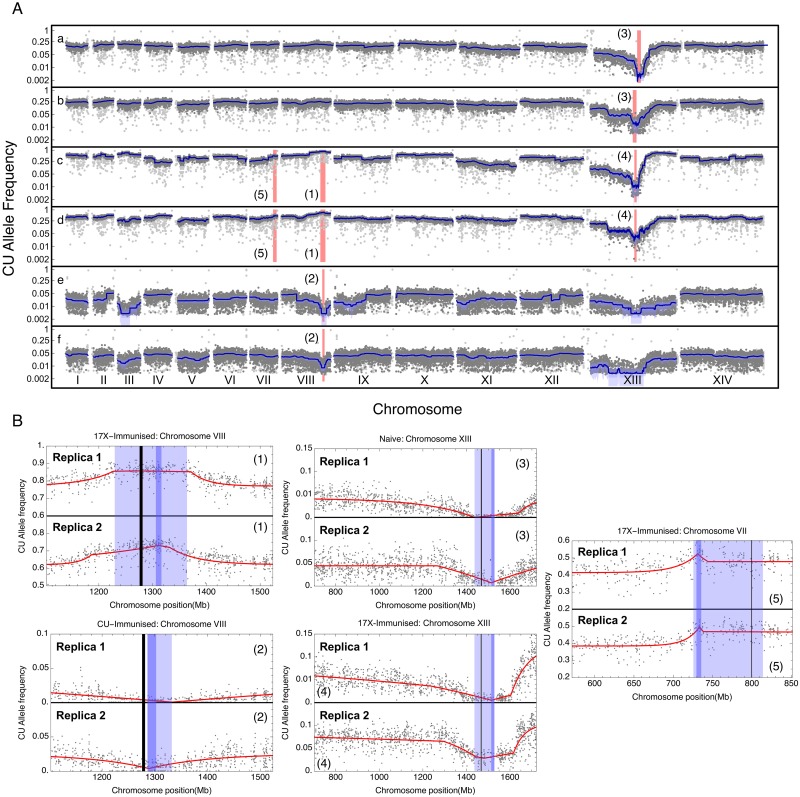
Genome-wide sequencing data. **(A)** Genome-wide *Plasmodium yoelii* CU allele frequency of two independent genetic crosses grown in (a,b) naïve mice, (c,d) 17X1.1pp immunized mice and (e,f) CU-immunized mice. Light gray dots represent observed allele frequencies. Dark gray dots represent allele frequencies retained after filtering. Dark blue lines represent a smoothed approximation of the underlying allele frequency; a region of uncertainty in this frequency, of size three standard deviations, is shown in light blue. A conservative confidence interval describing the position of an allele evolving under selection is shown via a red bar. Allele frequencies are shown in log scale. **(B)** Evolutionary models fitted to allele frequency data. Filtered allele frequencies are shown as gray dots, while the model fit is shown as a red line. Dark blue and light blue vertical bars show combined and conservative confidence intervals for the location of the selected allele as reported in Table 3. Numbers in parentheses equate figures with locations in **(A)**. A black vertical line shows the position of a gene of interest.

Of the final three putative loci, two were detected under multiple experimental conditions (Fig 3). When considering the combined largest intervals, a selective sweep was inferred at position 1,436–1,529 kb on Chromosome (Chr) XIII in replicate crosses grown in both non-immunized mice and 17X1.1pp-immunized mice, resulting from selection against CU-specific alleles at the target locus. A second sweep was inferred at position 1,229–1,364 kb on Chr VIII, detected in the parasite crosses grown in both CU and 17X1.1pp immunized mice, though not in the non-immunized mice. Here, selection pressure acted against different alleles according to the strain against which mice were immunized. The third sweep was detected at a locus between positions 725–814 kb on Chr VII. This event was only detected in mice replicates immunized with the 17X1.1pp strain, albeit that a consistent change in allele frequencies was also observed between replicas grown under these conditions (Fig 3B). The remaining loci (on Chrs VIII and XIII) were not consistently detected between replicates (S1 Table) and were thus considered to be non-significant.

### Potential target genes within the three main loci under selection

All the genes in the combined conservative intervals of the three main loci under selection are listed in S4–S6 Tables, along with annotation pertaining to function, structure, orthology with *P. falciparum* genes and Non-synonymous/Synonomous SNP (NS/S) ratio in the *P. falciparum* orthologue, which is calculated by the PlasmoDB website (6.2) based on SNP data from 202 individual strains. These include both laboratory strains and field isolates obtained from six collections (see Methods for more details). The locus associated with SSI on Chr VIII contains 41 genes. We considered the presence of either transmembrane (TM) domains or a signal peptides as necessary features of potential antigen-encoding genes. Only 16 genes met these criteria. Functional annotation indicated 10 likely candidates among these; eight genes described as “conserved *Plasmodium* proteins”, and two encoding RhopH2 and merozoite surface protein 1 (MSP1). Of these genes, the *P. falciparum* orthologue of *msp1* had the highest NS/S SNP ratio (8.43). MSP1 is a well characterized major antigen of malaria parasites that has formed the basis of several vaccine studies [24] and has been previously linked to SSI in *Plasmodium chabaudi* [10–12].

The locus under selection on Chr VII consists of 21 genes. Only seven contained TM domains and/or a signal peptide motif. Based on functional annotation, four of these could be potential targets for SSI. One of these genes, PY17X_0721800, encodes an apical membrane protein orthologous to *Pf34* in *P. falciparum*. This protein has recently been described as a surface antigen that can elicit an immune response [25]. Three conserved proteins of unknown function (PY17X_0720100, PY17X_0721500 and PY17X_0721600) also displayed potential signatures as target antigens.

The growth rate associated selected locus on Chr XIII contains 29 genes. In this case, the presence of TM domains or signal peptide motifs were not considered informative criteria. Only eight genes contained NS SNPs between the parental strains 17X1.1pp and CU according to the WGS data. Among these was a duffy binding protein, *Pyebl*. *Pyebl*, is a gene that has been previously implicated in growth rate differences between strains of *P. yoelii* [8, 26]. A single NS SNP was predicted from the WGS data in this gene. Due to the very high likelihood of its involvement based on previous work, this gene was considered for further analysis.

### Characterization of EBL as the major driver of growth rate differences through allelic replacement

**Fig 4:**
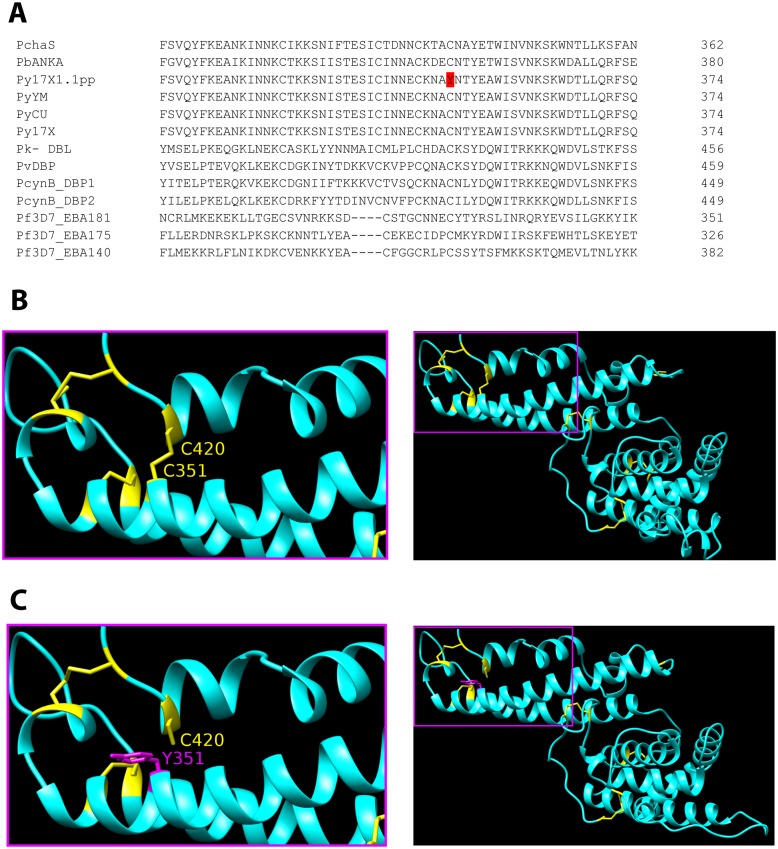
EBL Amino acid sequence alignment of various malaria species and *Plasmodium yoelii* strains, and predicted protein structure consequences of the C351Y polymorphism. **(A)** EBL orthologous and paralogous sequences from a variety of malaria species and *P. yoelii* strains were aligned using ClustalW. Only the amino acids surrounding position 351 are shown. The cysteine in positon 351 in *P. yoelii* is highly conserved across strains and species, with only strain 17X1.1pp bearing a C to Y substitution. PchAS: *Plasmodium chabaudi* AS strain; PbANKA: *Plasmodium berghei* ANKA strain; Py17X/17X1.1pp/CU/YM: *P. yoelii* 17X,17X1.1pp,CU,YM strains; Pk-DBLα/β/γ: *Plasmodium knowlesi* Duffy Binding Ligand alpha/beta/gamma (H strain); PvDBP: *Plasmodium vivax* Duffy Binding Protein (Sal-I strain);PcynB_DBP1/2: *Plasmodium cynomolgi* Duffy Binding Proteins 1/2 (B strain); Pf3D7_EBA140/175/181: *Plasmodium falciparum* Erythrocyte Binding Antigens 140/175/181 (3D7 strain). **(B)** Energy minimized homology model of the wild type *P. yoelii* (Py17XWT) Erythrocyte Binding Ligand (EBL). Inset depicts the disulfide bond between C351 and C420. (The protein is represented in cyan and the disulfide bonds are in yellow). **(C)** Energy minimized homology model of the mutant (C351Y) *P. yoelii* (Py17X1.1pp) Erythrocyte Binding Ligand (EBL). Inset depicts the lack of a disulfide bond between Y351 (substituted C351) and C420. (The protein is represented in cyan and the disulfide bonds are in yellow and Tyr351 [mutated] is represented in magenta).

Examining the *Pyebl* gene, Sanger capillary sequencing re-confirmed the existence in 17X1.1pp of an amino acid substitution (Cys >Tyr) at position 351 within region 2 of the encoded protein. When aligned against other *P. yoelii* strains and other *Plasmodium* species, this cysteine residue is highly conserved, and the substitution observed in 17X1.1pp was novel (Fig 4A). Crucially, no other polymorphisms were detected in the coding sequence of the gene, including in region 6, the location of the SNP previously implicated in parasite virulence in other strains of *P. yoelii* [8]. Structural modeling of the EBL protein in both wild-type and 17x1.1pp (C351Y) mutants predicted the abolition of a a disulphide bond between C351 and C420 in the mutant parasites that alters the tertiary structure of the receptor binding region of the ligand in these parasites (Fig 4B and 4C).

**Fig 5:**
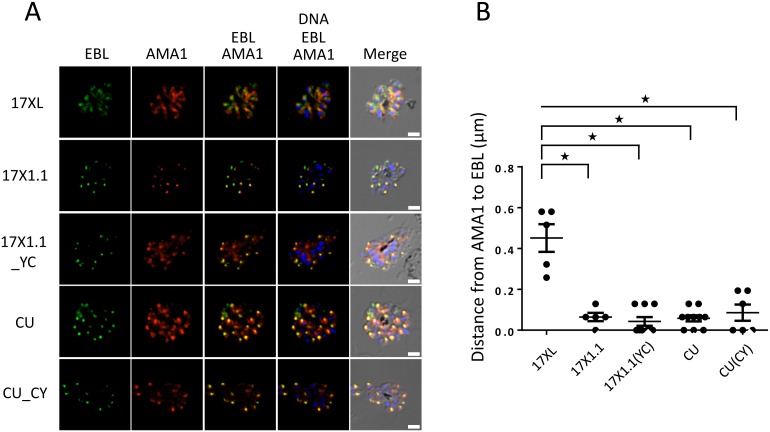
Localization of EBL. The C351Y polymorphism does not affect EBL subcellular localization in *Plasmodium yoelii*. **(A)** *P. yoelii* schizonts of wild type and transgenic parasite lines were incubated with fluorescent mouse anti-EBL serum, fluorescent rabbit anti-AMA1 serum, and DAPI nuclear staining. Colors indicate the localization of the *Pyebl*(green) and AMA-1 (red) proteins, as well as nuclear DNA (blue). 17XL: fast growing 17X clone previously shown to traffic EBL to the dense granules, not the micronemes, 17X1.1pp: 17x1.1pp strain, CU: CU strain, 17X1.1-351Y C: 17X1.1pp strain transfected with the CU allele for *Pyebl*, CU-351C Y: CU strain transfected with the 17X1.1pp allele of *Pyebl*. **(B)** The distance of EBL from AMA1 measured for five parasite strains and for 5–9 schizonts per strain; stars indicate p<0.01 using a Mann-Whitney U test. This indicates a shift in the location of *Pyebl* occurring in 17XL, but not in any other parasite lines.

The functional role of this polymorphism was verified by experimental means. In order to study the functional consequences of the polymorphism, the *Pyebl* alleles of slow growing CU and faster growing 17X1.1pp clones were replaced with the alternative allele (*i.e*. CU-EBL-351C>Y and 7x1.1pp-EBL-351Y>C), as well as with the homologous allele (*i.e*. CU-EBL-351C>C and 17x1.1pp-EBL-351Y>Y). The latter served as a control for the actual allelic swap, as the insertion of the plasmid for allelic substitution could potentially affect parasite fitness independently of the allele being inserted. To establish whether the C351Y substitution affected EBL localization, as was shown for the previously described region 6 mutation, Immunoflurescence Analysis (IFA) was performed. This revealed that, unlike the known mutation in region 6 [8], the EBL proteins of 17X1.1pp and CU were both found to be located in the micronemes (Fig 5 and S2 Fig).

**Fig 6:**
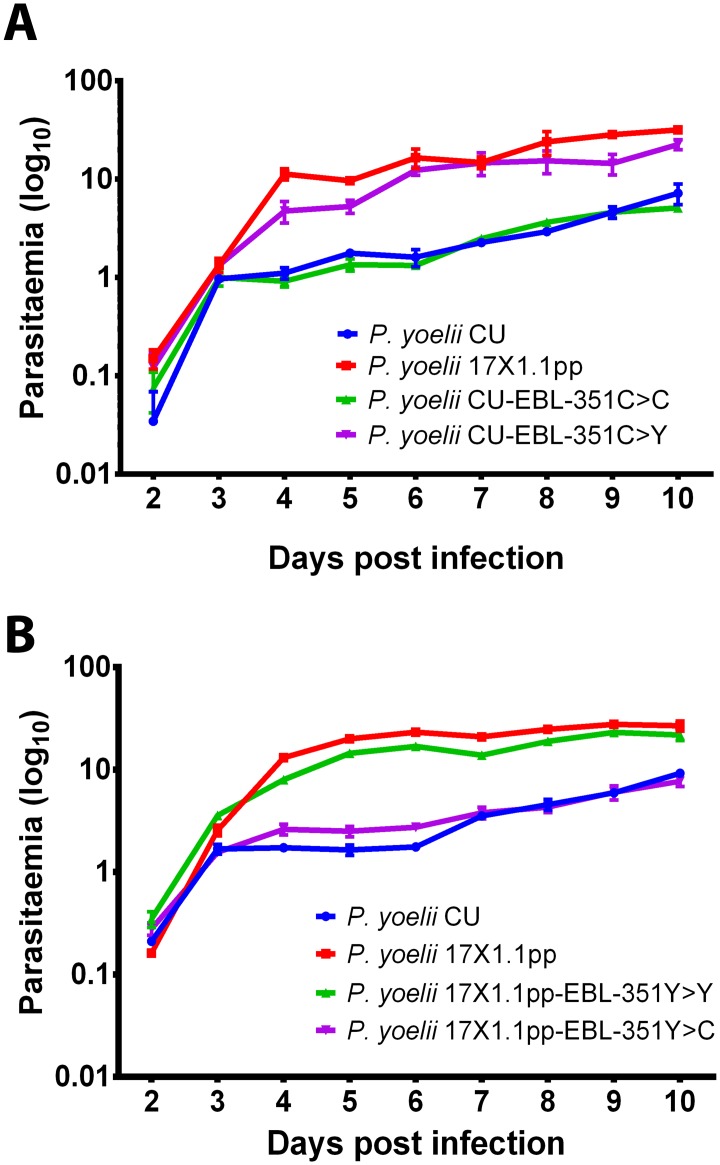
Site directed mutagenesis of *pyebl* AA position 351 reverses the phenotypes of parasites with slow and intermediate growth rates. **(A)** Growth rate of *P. yoelii* strains 17X1.1pp, CU and of the CU-strains transfected with either CU (CU-EBL-351C>C) or 17X1.1 (CU-EBL-351C>Y) *Pyebl* gene in CBA mice inoculated with 1x106 iRBCs on Day 0. **(B)** Growth rate of *P. yoelii* strains 17X1.1pp, CU and of the 17X1.1pp-strains transfected with either 17X1.1 (17X1.1pp-EBL-351Y>Y) or CU (17X1.1pp-EBL-351Y>C) *Pyebl* gene alleles in CBA mice inoculated with 1x106 iRBCs on Day 0. Transfection with the 17X1.1pp (EBL-351Y) allele produces a significantly increased growth rate in the CU strain (CU-EBL-351C>C vs CU-EBL-351C>Y: p <0.01, Two-way ANOVA with Tukey post-test correction) that is not significantly different from 17X1.1pp growth rate following transfection with its native allele (17X1.1pp-EBL-351Y>Y vs. CU-EBL-351C>Y: p >0.05, Two-way ANOVA with Tukey post-test correction). Conversely, transfection with the CU (EBA-351C) allele significantly reduces growth (17X1.1pp-EBL-351Y>Y vs 17X1.1pp-EBL-351Y>C: p <0.01, Two-way ANOVA with Tukey post-test correction) and produces a phenotype that is not significantly different from CU transfected with its own allele (CU EBL-351C>C vs 17X1.1pp-EBL-351Y>C: p >0.05, Two-way ANOVA with Tukey post-test correction).

Transgenic clones were grown in mice for 10 days alongside wild-type clones. Pair-wise comparisons between transgenic clones with the parental allele against transgenic clones with the alternative allele (that is CU-EBL-351C>C vs CU-EBL-351C>Y and 17x1.1pp-EBL-351Y>Y vs 17x1.1pp-EBL-351Y>C) showed that allele substitution could switch growth phenotypes in both strains (Fig 6A and 6B). This confirmed the role of the C351Y mutation as underlying the observed growth rate difference.

RNA-seq analysis revealed that transfected EBL gene alleles were expressed normally, (S3 Fig), thus indicating a structural effect of the polymorphism on parasite fitness, rather than an alteration in protein expression.

## Discussion

The development of LGS has facilitated functional genomic analysis of malaria parasites over the past decade. In particular, it has simplified and accelerated the detection of loci underlying selectable phenotypes such as drug resistance, SSI and growth rate [7, 8, 10]. Here we present a radically modified LGS approach that utilizes deep, quantitative WGS of parasite progenies and the respective parental populations, multiple crossing and mathematical modeling to identify loci under selection at ultra-high resolution. This enables the accurate definition of loci under selection and the identification of multiple genes driving selectable phenotypes within a very short space of time. This modified approach allows the simultaneous detection of genes or alleles underlying multiple phenotypes, including those with a multigenic basis.

Applying this modified LGS approach to study SSI and growth rate in *P. yoelii*, we identified three loci under selection that contained three strong candidate genes controlling both phenotypes. Two loci were implicated in SSI; the first time LGS has identified multigenic drivers of phenotypic differences in malaria parasites in a single experimental set-up. The strong locus under selection in Chr VIII, associated with the gene encoding MSP1, is consistent with existing knowledge of malaria immunity. The Chr VII locus, which includes the orthologue of *Pf34* as well as other potential unannotated antigens, underscores the power for hypothesis generation and gene detection of the LGS approach using multiple crosses.

Our approach also provided a genetic rationale for the difference in growth rate of the parental clones CU and 17X1.1pp. Phenotypically, this occurs due to the ability of 17X1.1pp to invade both reticulocytes and normocytes, while CU is restricted to reticulocytes [22]. Previously, differences in growth rates between strains of *P. yoelii* have been linked to a polymorphism in Region 6 of the *Pyebl* gene that alters its trafficking so that the protein locates in the dense granules rather than the micronemes [8, 26]. In the case of 17x1.1pp however, direct sequencing of the *Pyebl* gene revealed a previously unknown SNP in region 2, the predicted receptor-binding region of the protein, with no polymorphism in region 6. Consistent with this, the EBL protein of 17X1.1pp was shown to be located in the micronemes, indicating that protein trafficking was unaffected by the region 2 substitution. Allelic replacement of the parasite strains with the alternative allele resulted in a switching of the growth rate to that of the other clone, thus confirming the role of the substitution.

Region 2 of the *Pyebl* orthologues of *P. falciparum* and *Plasmodium vivax* [27–29] are known to interact with receptors on the red blood cell (RBC) surface. Furthermore, the substitution falls within the central portion of the region, which has been previously described as being the principal site of receptor recognition in *P. vivax* [29]. Wild-type strains of *P. yoelii* (such as CU) preferentially invade reticulocytes but not mature RBCs, whereas highly virulent strains are known to invade a broader repertoire of RBCs [30]. Further structural and functional studies are required to elucidate how the polymorphism described here enables mutant parasites to invade a larger repertoire of erythrocytes than wild type parasites. We show that the cysteine residue at position 351 in EBL forms a disulphide bond with a cysteine at position 420, and that this is abolished following the C351Y substitution, altering the tertiary structure of the binding region. This leads to the possibility that such an alteration of the shape of the binding domain may enable the ligand to bind to a larger repertoire of receptors.

LGS with multiple crosses offers a powerful and rapid methodology for identifying genes or non-coding regions controlling important phenotypes in malaria parasites and, potentially, in other apicomplexan parasites. Through bypassing the need to clone and type hundreds of individual progeny, and by harnessing the power of genetics, genomics and mathematical modeling, genes can be linked to phenotypes with high precision in a matter of a few months, rather than years. Here we have demonstrated the ability of LGS to identify multiple genetic polymorphisms underlying two independent phenotypic differences between a pair of malaria parasite strains; growth rate and SSI. This methodology has the potential power to identify the genetic components controlling a broad range of selectable phenotypes, and can be applied to studies of drug resistance, transmissibility, virulence, host preference, *etc*., in a range of apicomplexan parasites that are amenable to genetic crossing.

The applicability of the approach to human malaria species has been recently demonstrated: the original LGS approach was successfully applied to study *P. falciparum* immune evasion in mosquitoes *in vivo* [31], while we recently tested its applicability *in vitro* to detect loci under selection following antifolate drug treatment and *in vitro* growth rate competition. With the advent of humanized mice that are able to support the complete malaria life cycle, the generation of new genetic crosses between strains of human malaria has become more feasible, as recently demonstrated [32]. With the ability to maintain these crosses without the need of simian hosts, application of a broader range of selection pressures (excluding, for now, selection mediated by the presence of a complete immune response) is now more feasible *in vivo*, thus extending the application of the LGS approach to medically relevant malaria species.

## Materials and methods

Full and complete details of the mathematical methods are given in S1 Appendix, Supplementary Mathematical Methods.

### Ethics statement

Laboratory animal experimentation was performed in strict accordance with the Japanese Humane Treatment and Management of Animals Law (Law No. 105 dated 19 October 1973 modified on 2 June 2006), and the Regulation on Animal Experimentation at Nagasaki University, Japan. The protocol was approved by the Institutional Animal Research Committee of Nagasaki University (permit: 1207261005–2).

### Parasites, mice and mosquitoes

*Plasmodium yoelii* CU (with slow growth rate phenotype) and 17X1.1pp (with intermediate growth rate phenotype) strains [33] were maintained in CBA mice (SLC Inc., Shizuoka, Japan) housed at 23°C and fed on maintenance diet with 0.05% para-aminobenzoic acid (PABA)-supplemented water to assist with parasite growth. *Anopheles stephensi* mosquitoes were housed in a temperature and humidity controlled insectary at 24°C and 70% humidity, adult flies were maintained on 10% glucose solution supplemented with 0.05% PABA.

### Testing parasite strains for growth rate and SSI

*Plasmodium yoelii* parasite strains were typed for growth rate in groups of mice following the intravenous inoculation of 1 × 10^6^ iRBCs of either CU, 17X1.1pp or transfected clones per mouse and measuring parasitaemia over 8–9 days. In order to verify the existence of SSI between the CU and 17X1.1pp strains, groups of five mice were inoculated intravenously with 1 × 10^6^ iRBCs of either CU or 17X1.1pp parasite strains. After four days, mice were treated with mefloquine (20mg/kg/per day, orally) for four days to remove infections. Three weeks post immunization, mice were then challenged intravenously with 1 × 10^6^ iRBCs of a mixed infection of 17X1.1pp and CU parasites. A group of five naïve control mice was simultaneously infected with the same material. After four days of growth 10 *μl* of blood were sampled from each mouse and DNA extracted.

Strain proportions were then measured by Quantitative Real Time PCR using primers designed to amplify the *msp1* gene [34]. All measurements were plotted and standard errors calculated using the Graphpad Prism software (v6.01) (http://www.graphpad.com/scientific-software/prism/). Wilcoxon rank sum tests with continuity corrections were used to measure the SSI effect, and were performed in R [35]. Linear mixed model analyses and likelihood ratio tests to test parasite strain differences in growth rate were performed on log-transformed parasitaemia by choosing parasitaemia and strain as fixed factors and mouse nested in strain as a random factor, as described previously [22]. Pair-wise comparisons of samples for the transfection experiments were performed using multiple 2-way ANOVA tests and corrected with a Tukey’s post-test in Graphpad Prism software (v6.01).

#### Preparation of genetic cross

*Plasmodium yoelii*. CU and 17X1.1pp parasite clones were initially grown separately in donor mice. These parasite clones were then harvested from the donors, accurately mixed to produce an inoculum in a proportion of 1:1 and inoculated intravenously at 1 × 10^6^ infected red blood cells (iRBCs) per mouse into a group of CBA mice. Three days after inoculation, the presence of gametocytes of both sexes was confirmed microscopically and mice were anesthetized and placed on a mosquito cage containing 400 female *A. stephensi* mosquitoes six to eight days post emergence. Mosquitoes were then allowed to feed on the mice without interruption. Seven days after the blood meal, 10 female mosquitoes from this cage were dissected to examine for the presence of oocysts in mosquito midguts. Seventeen days after the initial blood meal, the mosquitoes were dissected, and the salivary glands (containing sporozoites) were removed. The glands were placed in 0.2–0.4 mL volumes of 1:1 foetal bovine serum/Ringer’s solution (2.7 mM potassium chloride, 1.8 mM calcium chloride, 154 mM sodium chloride) and gently disrupted to release sporozoites. The suspensions were injected intravenously into groups of CBA mice in 0.1 mL aliquots to obtain blood stage *P. yoelii* CU17X1.1pp cross progeny. Three days after inoculation with sporozoites, blood stage *P. yoelii* CU17X1.1pp cross progeny parasitized-RBC (pRBC) were harvested.

Two independent genetic crosses between CU and 17X1.1pp were produced. In the first cross, 150 mosquitoes were allowed to feed on mice inoculated 3 days previously with a 50:50 mixture of the two parental strains. Seven days later, a sub sample of mosquitoes (n = 25) were dissected for oocyst detection. In this case, 90% of mosquitoes were infected, with an average burden of 87 oocysts per mosquito. Given that 50% of the oocysts are expected to be the products of selfing (*i.e*. CU male gametes fertilizing CU female gametes, and 17X1.1pp male gametes fertilizing 17x1.1pp female gametes), and that the remaining 50% of oocysts resulting from cross-strain fertilization would each produce four recombinant progeny types, we estimate that this cross resulted in the inoculation of 15,660 recombinant progeny types to recipient mice on day 21 post-mosquito feed, when 100 mosquitoes were dissected and the sporozoites removed from the salivary glands for inoculation. For the second cross, which followed the same protocol, 60% of mosquitoes were infected with an average oocyst burden of 77 oocysts per mosquito, leading to an estimated 9240 recombinants in the cross inoculation.

#### Selection of uncloned cross progeny for linkage group selection analysis

For immune selection, mice immunized with blood stage parasites of either *P. yoelii* CU or 17X1.1pp through exposure and drug cure (as above) were inoculated intravenously with 1 × 10^6^ parasitized-RBC (pRBC) of the uncloned cross progeny, as described above. The resulting infections were followed by microscopic examination of thin blood smears stained with Giemsa’s solution.

#### DNA and RNA isolation

Parental strains and growth rate- or immune-selected recombinant parasites were grown in naïve mice. Parasite-infected blood was passed through a single CF11 cellulose column to deplete host leukocytes, and the genomic DNA (gDNA) was isolated from the saponin-lysed parasite pellet using DNAzol reagent (Invitrogen, Carlsbad, CA, USA) according to the manufacturer’s instructions. For RNA isolation, a schizont-enriched fraction was collected on a 50% Nycodenz solution (Sigma Aldrich) and total RNA was then isolated using TRIzol (Invitrogen).

#### Whole genome re-sequencing and mapping

*Plasmodium yoelii* genomic DNA was sequenced using paired end Illumina reads (100 bp), which are available at the European Nucleotide Archive (ENA: PRJEB15102). The paired-end Illumina data were first quality-trimmed using Trimmomatic [36]. Illumina sequencing adaptors were then removed from the sequences. Following that, trailing bases from both the 5’ and 3’ ends with less than Q20 were trimmed. Lastly, reads with an average base quality of less than Q20 within a window size of four bases were discarded. Only read pairs where both reads were retained after trimming were used for mapping with BWA [37] version 0.6.1 using standard options onto the publicly available genome of *P. yoelii* 17X strain (May 2013 release; ftp://ftp.sanger.ac.uk/pub/pathogens/Plasmodium/yoelii17X/version_2/May_2013/). The SAM alignment files were converted to BAM using Samtools [38]. Duplicated reads were marked and removed using Picard (http://picard.sourceforge.net).

#### SNP calling

The Python script [18] used to determine SNP functions as a wrapper for SAMtools mpileup and SNP calls based on mapping quality and Phred base quality scores. In this experiment the values were set at 30 for mapping quality and 20 for base quality. Also, since the *P. yoelii* genome is haploid and the parental strains are clonal, only SNPs where the proportion of the major non-reference allele was more than 80% were retained, to exclude possible sequencing errors or genuine but uninformative SNPs. The script produces a tab-delimited, human readable table that shows the total number of reads for each of the four possible nucleotides at each SNP. SNPS were called on both parental strains. CU SNPs were then filtered against the 17X1.1pp SNPs to remove any shared SNP calls. The remaining CU SNPs were then used as reference positions to measure the number of reads for each nucleotide in the genetic crosses produced in this study through another Python script [18]. This script produced a final table consisting of read counts for each nucleotide of the original CU SNPs in every sample.

### Mathematical methods for the identification of loci under growth rate and immune selection

SNP frequencies were processed to filter potential misalignment events. We note that, during the cross, a set of individual recombinant genomes are generated. Considering the individual genome *g*, we define the function *a*_*g*_(*i*) as being equal to 1 if the genome has the CU allele at locus *i*, and equal to 0 if the genome has the 17X1.1pp allele at this locus. In any subsequent population of *N* individuals, the allele frequency q(*i*) at locus *i* can then be expressed as

$$\begin{array}{c} q\left(i\right)=\frac{1}{N}\;\sum_{g}\;n_{g}a_{g}\left(i\right) \end{array}$$ (1)

for some set of values *n*_*g*_, where *n*_*g*_ is the number of copies of genome *g* in the population.

To filter the allele frequencies, we note that each function *a*_*g*_(*i*) changes only at recombination points in the genome *g*. As such, *q*(*i*) should change relatively smoothly with respect to *i*. Using an adapted version of code developed for the inference of subclones in populations [39], we therefore modeled the reported frequencies *q*(*i*) as being (beta-binomially distributed) emissions from an underlying diffusion process (denoted by *x*(*i*)) along each chromosome, plus uniformly distributed errors, using a hidden Markov model to infer the variance of the diffusion process, the emission parameters, and an error rate. A likelihood ratio test was then applied to identify reported frequencies that were inconsistent with having been emitted from the inferred frequency *x*(*i*) at locus i relative to having been emitted from an inferred global frequency distribution fitted using the Mathematica package via Gaussian kernel estimation to the complete set of values {*x*(*i*)}; this test filters out reported frequencies potentially arising from elsewhere in the genome.

Next, the above logic was extended to filter out clonal growth. In the event that a specific genome *g* is highly beneficial, this genome may grow rapidly in the population, such that *n*_*g*_ becomes large. Under such circumstances the allele frequency *q*(*i*) gains a step-like quality, mirroring the pattern of *a*_*g*_(*i*). Such steps may potentially mimic selection valleys, confounding any analysis. As such, a jump-diffusion variant of the above hidden Markov model was applied, in which the allele frequency can change either through a diffusion process or via sudden jumps in allele frequency, modeled as random emissions from a uniform distribution on the interval [0, 1]. For each interval (*i*,*i* + 1) the probability that a jump in allele frequency had occurred was estimated. Where potential jumps were identified, the allele frequency data were split, such that analyses of the allele frequencies did not span sets of alleles containing such jumps. The resulting segments of genome were then analyzed under the assumption that they were free of allele frequency change due to clonal behavior.

Inference of the presence of selected alleles was performed using a series of methods. In the absence of selection in a chromosome, the allele frequency is likely to remain relatively constant across each chromosome. A ‘non-neutrality’ likelihood ratio test was applied to each contiguous section of genome, calculating the likelihood difference between a model of constant frequency *x*(*i*) and the variable frequency function *x*(*i*) inferred using the jump-diffusion model. Next, an inference was made of the position of the allele potentially under selection in each region. Under the assumptions that selection acts for an allele at locus *i*, and that the rate of recombination is constant within a region of the genome, previous work on the evolution of cross populations [19, 20] can be extended to show that the allele frequencies within that region of the genome at the time of sequencing are given by

$$x(i)=x+\Delta_{x}$$ (2)

$$x(j)=[X+\frac{1}{2}\left(1-X\right)(1+e^{-\rho \Delta_{ij}}]\;x+[\frac{1}{2}X\left(1-e^{-\rho \Delta_{ij}}\right)]\;\left(1-x\right)+\Delta_{x}$$ (3)

for each locus *j* not equal to *i*, where *X* is the CU allele frequency at the time of the cross, *ρ* is the local recombination rate, Δ_*ij*_ is the distance between the loci *i* and *j*, *x* is an allele frequency, and Δ_*x*_ describes the effect of selection acting upon alleles in other regions of the genome. A likelihood-based inference was used to identify the locus at which selection was most likely to act. In regions for which the ‘non-neutrality’ test produced a positive result for data from both replica crosses, and for which both the inferred locus under selection, and the direction of selection acting at that locus were consistent between replicas, an inference of selection was made.

For regions in which an inference of selection was made, an extended version of the above model was applied, in which the assumption of locally constant recombination rate was relaxed. Successive models, including an increasing number of step-wise changes in the recombination rate, were applied, using the Bayesian Information Criterion [40] for model selection. A model of selection at two loci within a region of the genome was also examined. Given an inference of selection, a likelihood-based model was used to derive confidence intervals for the position of the locus under selection.

### Information on genes in identified loci under selection

For each combined conservative interval of relevant loci under selection, genes were listed based on the annotation available in version 6.2 of PlasmoDB and verified against the current annotation (release 26). For each gene, information on predicted transmembrane domains, signal peptides and *P. falciparum* orthologues. For the *P. falciparum* orthologues, the NS/S SNP ratios were obtained from PlasmoDB, based on the count of synonymous and non-synonymous SNPs found in 202 individual strains collected from 6 data sets stored on the website. More details on the data sets can be found at the following link: https://goo.gl/lUwKn1.

### Plasmid construction to modify *P. yoelii ebl* gene locus

All primer sequences are given in Supplementary S7 Table. Plasmids were constructed using MultiSite Gateway cloning system (Invitrogen).

#### PCR amplification and sequencing of the *Pyebl* gene

The *Pyebl* gene was PCR-amplified from gDNA using KOD Plus Neo DNA polymerase (Toyobo, Japan) with specific primers designed based on the *e*bl sequence in PlasmoDB (PY17X_1337400). *Pyebl* sequences of CU and 17X1.1pp strains were determined by direct sequencing using an ABI PRISM 310 genetic analyzer (Applied Biosystems) from PCR-amplified products. Sequences were aligned using online sequence alignment software Clustal Omega (https://www.ebi.ac.uk/Tools/msa/clustalo/) provided by EMBL-EBI.

#### Plasmid construction to modify the *Pyebl* locus

attB-flanked *ebl* gene products, attB12-*Py*CU-EBL.ORF and attB12-Py17X1.1pp-EBL.ORF, were generated by PCR-amplifying both *P. yoelii* CU and *P. yoelii* 17X1.1pp *ebl* gene with yEBL-ORF.B1F and yEBL-ORF.B2R primers. attB-flanked *ebl*-3U (attB41-*Py*CU-EBL-3U and attB41-*Py*17X1.1pp-EBL-3U) was similarly generated by PCR-amplifying *P. yoelii* gDNA with yEBL-3U.B4F and yEBL-3U.B1R primers. attB12-*Py*CU-EBL.ORF and attB12-*Py*17X1.1pp-EBL.ORF were then subjected to a separate BP recombination with pDONR 221 (Invitrogen) to yield entry plasmids, pENT12-PyCU-EBL.ORF and pENT12-Py17X1.1pp-EBL.ORF, respectively. attB41-PyCU-EBL-3U and attB41-Py17X1.1pp-EBL-3U fragments were also subjected to independent BP recombination with pDONR P4-P1R (Invitrogen) to generate pENT41-*Py*CU-EBL-3U and pENT41-*Py*17X1.1pp-EBL-3U, respectively. All BP reactions were performed using the BP Clonase II enzyme mix (Invitrogen) according to the manufacturer’s instructions. To change *P. yoelii* CU *ebl* gene nucleotide 1052G to 1052A (351Cys to 351Tyr), pENT12-PyCU-EBL.ORF entry clone was modified using KOD-Plus-Mutagenesis Kit (TOYOBO) with primers P1.F and P1.R to yield pENT12-*Py*CU-EBL.ORF-C351Y. pENT12-*Py*17X1.1pp-EBL.ORF was also modified from 1052A to 1052G (351Tyr to 351Cys) using primers P2.F and P1.R to yield pENT12-*Py*17X1.1pp-EBL.ORF-Y351C. pHDEF1-mh that contains a pyrimethamine resistant gene selection cassette [41] (a gift from Hernando del Portillo) was digested with *SmaI* and *ApaI* to remove PfHRP2 3’ UTR DNA fragment, cohesive end was blunted, and a DNA fragment containing ccdB-R43 cassette and *P. berghei* DHFR-TS 3’ UTR that was amplified from pCHD43(II) [42] with primers M13R.F3F and PbDT3U.F3R was ligated to generate pDST43-HDEF-F3. pENT12-*Py*CU-EBL.ORF-C351Y and pENT12-*Py*17X1.1pp-EBL.ORF-Y351C entry plasmids were each separately subjected to LR recombination reaction (Invitrogen) with a destination vector pDST43-HDEF-F3, pENT41-PyCU-EBL-3U or pENT41-*Py*17X1.1pp-EBL-3U and a linker pENT23-3Ty1 vector to yield replacement constructs pREP-*Py*CU-EBL-C351Y and pREP-*Py*17X1.1pp-EBL-Y351C, respectively. Control constructs pREP-*Py*CU-EBL-C351C and pREP-*Py*17X1.1pp-EBL-Y351Y were also prepared in a similar manner. These LR reactions were performed using the LR Clonase II Plus enzyme mix (Invitrogen) according to the manufacturer’s instructions.

### Phenotype analysis

To assess the course of infection of wild type and transgenic parasite lines, 1 × 10^6^ pRBCs were injected intravenously into five 8-week old female CBA mice for each parasite line. Since the 17X1.1p and CU-recipient strains were transfected on separate occasions, the transgenic lines were tested separately. Thin blood smears were made daily, stained with Giemsa’s solution, and parasitaemias were examined microscopically.

#### RNA-seq

Whole blood from mice infected with *P. yoelii* on day 5 post-infection were host WBC depleted and saponin lysed to obtain the parasite pellet. Total RNA was extracted using TRIzol reagent. Strand-specific RNA sequencing was performed from total RNA using TruSeq Stranded mRNA Sample Prep Kit LT according to manufacturer’s instructions. Libraries were sequenced on an Illumina HiSeq 2000 with paired-end 100bp read chemistry and are publicly available at the European Nucleotide Archive (ENA: PRJEB15102). RNA-seq reads were mapped onto *P. yoelii* 17X version 2 from GeneDB (http://www.genedb.org) using TopHat 2.0.13 [43] and visualized using Artemis genome visualization tool [44].

#### Indirect immunofluorescence assay

Schizont-rich whole blood was obtained from *P. yoelii* infected mouse tail and prepared air-dried thin smears on glass slides. The smears were fixed in 4% paraformaldehyde containing 0.0075% glutaraldehyde (Nacalai Tesque) in PBS at room temperature (RT) for 15 min, rinsed with 50 mM glycine (Wako) in PBS. Samples were permeabilized with 0.1% Triton X–100 (Calbiochem) in PBS for 10 min, then blocked with 3% BSA (Sigma) in PBS at RT for 30 min. Next, samples were immunostained with primary antibodies using mouse anti–PyEBL [26] (final 1:500) and Rabbit anti–PyAMA1 [45] (a gift from Takafumi Tsuboi, final concentration 1:500) at 37°C for 1 h. This was followed by 3 washes with PBS then incubation with Alexa Fluor 488 goat anti–mouse and Alexa Fluor 594 goat anti–rabbit antibodies (Invitrogen; final 1:1000) in 3% BSA in PBS at 37°C for 30 min. Parasite nuclei were stained with 4’, 6-diamidino-2-phenylindole (DAPI; Invitrogen, final 0.2 *μ*g/mL). Stained parasites were mounted with Prolong Gold antifade reagent (Invitrogen). Slides were visualized using a fluorescence microscope (Axio imager Z2; Carl Zeiss) with 100x oil objective lens (NA 1.4, Carl Zeiss). Images were captured using a CCD camera (AxioCam MRm; Carl Zeiss) and imaged using AxioVision software (Carl Zeiss). Mann-Whitney U tests were performed using Graphpad Prism software (v6.01).

### Structural modeling of PyEBL protein in wild-type and mutant parasites

Since the atomic structures of EBL protein of *P. yoelii* Wild Type: (Py17X-WT) and its mutant *P. yoelii* (C351Y): (Py17X1.1pp) are not known, homology models were generated. The homology models were generated using *P. vivax* Duffy Binding Protein (PvDBP) atomic structure (PDB ID: 3RRC, [46] with the Swiss-Model server (https://swissmodel.expasy.org) [47–50]. The homology models showed maximum amino acid sequence homology of 32% with Py17X-WT EBL, compared to another homologous protein *P. falciparum* Erythrocyte Binding Antigen 140 (PfEBA-140/BAEBL) (PDB ID: 4GF2, [51], that had 26% sequence homology. These models were then subsequently stabilized by minimizing their energies for at least 10 times each, to attain reasonably well equilibrated structures using the YASARA server (www.yasara.org).

The prediction of disulfide bonds in our homology models were performed using DISULFIND (http://disulfind.dsi.unifi.it) [52–55]. Our analysis showed high probability of disulfide bond formation by this Cys351 residue. Confirming that C351 is a potential residue for forming a disulfide bond, the energy minimized stable homology models were subjected to Disulfide bond visualization to check whether the Cys351 is involved in any disulfide bond formation with any other Cys and what is the effect of the C351Y substitution.

The homology models along with their disulfide bonds were visualized (Fig 4B and 4C) and the images were obtained using the “Disulfide by Design 2.0” server (http://cptweb.cpt.wayne.edu) [56].

### Code

Code used in this project is available online from https://github.com/cjri/LGSmalaria

## Supporting information

S1 Appendix Supplementary mathematical methods. (PDF)

S1 Fig Parasitaemias after immune challenges. (PDF)

S2 Fig Intracellular localization of EBL in parasite strains CU, 17XL, 17X1.1pp and in transfected parasites CU(CY) and 17X1.1pp(YC). (PDF)

S3 Fig Expression of *Pyebl* alleles in both wild type (WT) and transfected strains. (PDF)

S1 Table Selected alleles identified by the SDR model. (PDF)

S2 Table Bayesian Information Criterion (BIC) values for varying models for candidate regions of the genome, within each replica, calculated under different models. (PDF)

S3 Table Inferred recombination rates from driver models. (PDF)

S4 Table List of genes contained within the mathematically defined confidence intervals (725,528–813,866 bp) of the locus under selection on chromosome 7. (PDF)

S5 Table List of genes contained within the mathematically defined confidence intervals (1,229,582–1,363,920 bp) of the locus under selection on chromosome 8. (PDF)

S6 Table List of genes contained within the mathematically defined confidence intervals (1,436,717–1,528,275 bp) of the locus under selection on chromosome 13. (PDF)

S7 Table PCR primers used to generate constructs for transfection experiments. (PDF)
