# Supplementary material for: Rapid identification of genes controlling virulence and immunity in malaria parasites
Source: PLoS Pathog. 2017 Jul 12;13(7):e1006447. doi: 10.1371/journal.ppat.1006447 (PMC5507557; doi:10.1371/journal.ppat.1006447)
Supplement: S1 Appendix — (PDF) [file ppat.1006447.s001.pdf]

Rapid Identification of Genes Controlling Virulence and Immunity in Malaria Parasites

Hussein M Abkallo, Axel Martinelli, Megumi Inoue, Abhinay Ramaprasad, Jesse Gitaka, Jianxia Tang, Kazuhide Yahata, Augustin Zoungrana, Hayato Mitaka, Arita Acharjee, Partha P Datta, PaulHunt, Richard Carter, Osamu Kaneko, Ville Mustonen, Christopher JR Illingworth, Arnab Pain, Richard Culleton,

Supporting Information

S1 Appendix. Supplementary Mathematical Methods.

Filtering of allele frequency data: diffusion model

Allele frequency data were filtered using a likelihood ratio in an effort to remove sites where alleles had been mapped to the wrong genomic location. Given the structure of the genetic cross, the allele frequency is expected to change incrementally with small changes in genetic location. We therefore generated a smoothed representation of the underlying allele frequencies. For each genetic locus i, with read depth N_i, we denote the read count of CU alleles by n_i, and the true underlying CU allele frequency by x_i. We then suppose that, with some probability $1-r$, n_i was drawn from a beta-binomial distribution $Beta(N_i, \alpha, \beta)$, where $\alpha = cx_i$, and $\beta = c(1-x_i)$, for some unknown parameter c, while with probability r, n_i resulted from a mapping error, being drawn from the uniform distribution $U(0, N_i)$. We further supposed that changes in the true allele frequencies between nearby loci are small, being represented by a diffusion process:

$$x_{i+1} = x_i + \mathcal{N}(0, s\sqrt{\Delta_{i,i+1}}), \tag{1}$$

in which the difference between subsequent allele frequencies is normally distributed with zero mean and standard deviation proportional to s times the square root of the distance between the segregating sites (reflecting boundaries were used to keep x_i within the interval [0,1]). Given this model, a forward-backward algorithm was used to identify maximum likelihood values for r, c, and s. Our algorithm gave a posterior distribution for each of the x_i; we calculated the mean of this distribution to obtain approximations $\hat{x}_i$ for each locus.

A likelihood ratio test was then applied to exclude frequencies of alleles that were likely to have been mapped to the wrong location in the genome. Expressed in terms of the above parameters, the likelihood L_1 that an allele frequency belonged to the genomic region with which it had been associated was estimated as

$$L_1 = \binom{N_i}{n_i} \frac{B(n_i + c\,\hat{x}_i, N_i - n_i + c\,(1-\hat{x}_i))}{B(c\,\hat{x}_i,\, c\,(1-\hat{x}_i))} \tag{2}$$

where $B(a,b)$ is the beta function. In contrast to this, a mismapped read could arise from anywhere in the genome. Using the Mathematica software package, a smooth kernel distribution was fitted to the set $\{\hat{x}_i\}$, of all observed frequencies genome-wide, obtaining the probability density function P for this distribution. The likelihood L_2 was then calculated as

$$L_2 = \mathcal{P}(\hat{x}_i) \tag{3}$$

Data from loci for which the log ratio $\log(L_1/L_2) < -10$ in at least one of the replicates were excluded from further analysis in all datasets.

Particular care was taken with alleles mapped to regions at the ends of chromosomes. Firstly, small sets of isolated allele frequencies, occurring at the ends of chromosomes, were excluded from the analysis. Loci within each chromosome were partitioned into subsets, separated by gaps of at least 20kb in which no SNPs were observed. Subsets of fewer than 10 isolated loci at the ends of chromosomes were removed from the data.

Jump diffusion analysis

From visual inspection of the data, occasional apparent discontinuities were seen, at which the observed allele frequency changed substantially between adjacent SNPs. These jumps could occur either from the growth of a clone, or clones, with near-identical genomes, in the experimental population [1], or alternatively through some gross misalignment of data, whereby regions some distance apart in the genome were placed together.

The location of significant jumps in the allele frequency was inferred by modeling the observed data as being generated by a jump-diffusion process, fitting a set of frequencies x_i to the observations which change either smoothly, according to a diffusion model as described above, or through sudden changes to different, arbitrary frequencies. Specifically, x_i was modeled as changing via the equations $x_{i+1} = x_i + \mathcal{N}(0, s\sqrt{\Delta_{i,i+1}})$ with probability $(1-p)^{\Delta_{i,i+1}}$ and $x_{i+1} \sim \mathcal{U}(0,1)$ with probability $1-(1-p)^{\Delta_{i,i+1}}$, where the value p represents the probability per base of a jump in allele frequency. Parameters were inferred as above, with the addition of the value p. The beta-binomial coefficient c was fixed as the value inferred for each dataset from the previous calculation. Due to the earlier filtering steps, applied above, the inferred error rate r was less than 10^{-10} for each set of allele frequencies, so was removed from the model. For each locus i the posterior probability p_i that a jump occurred at i was calculated.

Loci with posterior jump probabilities greater than 1% are listed in Table 1. Three of these loci, towards the ends of chromosomes, were conserved between replicates, being seen in both of the 17X-immunised datasets, a jump in chromosome XIV being observed in both naïve replicates as well. Such consistency in the location of jumps between replica experiments is highly improbable if they occur independently; we supposed these jumps to result from misalignment errors, or errors in the genome reference sequence. Alleles further towards the end of each chromosome than these jumps were removed from consideration in all datasets.

Other loci at which jumps were inferred were only seen in the first replicate experiment, primarily in the 17X-immunised data, but also in the naïve dataset. This result is consistent with the existence of clonal growth in the first replica experiment, some of it occurring before the separation of parasite populations into naïve and selected groups. The reduced number of jumps in the naïve and CU-immunised cases may be explained by a difficulty in inference; due to pervading selection for 17X alleles, the mean allele frequency in these two populations is generally close to 0, reducing the magnitude of observed jumps in frequency.

In order to fit models of continuous allele frequency change to the observed frequency data, chromosomes were subdivided into smaller regions at the location of potential jumps, such that the frequencies within each region under analysis changed in a continuous manner.

Likelihood models

Regions of the genome containing alleles under selection were identified using a likelihood-based modeling framework. Given a model M describing allele frequencies

after selection, the model parameters were optimised to identify the maximum likelihood fit between the model, and the observed frequencies in a genomic region, using the noise model learnt in the diffusion model above:

$$\log L^M = \sum_i \log \binom{N_i}{n_i} \frac{B(n_i + c\,x_i, N_i - n_i + c\,(1-x_i))}{B(c\,x_i, c\,(1-x_i))} \tag{4}$$

In order to distinguish between likelihoods generated from models with differing numbers of parameters, the Bayesian Information Criterion (BIC) was used. For a given model fit to the data, the BIC value is given by

$$\mathrm{BIC} = -2L + k\log(n) \tag{5}$$

where k is the number of model parameters, and n is the number of loci to which the model was fitted. In any comparison between models, the model giving the lowest BIC value was selected.

A variety of models were applied, modeling changes in the allele frequency over time between the beginning of the experiment and the time of sequencing. A neutral model assumed that no alleles were under selection. A single driver model (SD) assumed that a single allele, or “driver” within the region was under selection. These standard models assumed a locally-constant recombination rate; extensions of the single-driver model allowed for one (SDR), two (SD2R), or three (SD3R) changes in recombination rate within the local region. Further comparison was made to the jump-diffusion (J-D) model described above, in which a smooth line was fitted directly to the allele frequencies; the jump-diffusion model is by its definition a very good fit to the data.

Identification of non-neutral regions of the genome

Non-neutral regions of the genome were identified according to two characteristics. Firstly, we note that, if no alleles in a given region of the genome are under selection, the allele frequencies in this region may still change during the experiment, due to selection acting upon pure genotypes during the cross, but will do so in a uniform way, plus noise. However, if a single allele is under selection, this will result in local variation in the observed allele frequencies, according to the pattern of a selective sweep [2]). As such, regions of the genome were tested for deviation from neutrality; comparing the log likelihoods generated by the neutral and J-D models. The “non-neutrality score” S for a region of the genome g taken from replica r, was defined as

$$S_{r,g} = \frac{L^{\mathrm{JD}}_{r,g} - L^{\mathrm{neutral}}_{r,g}}{n_g} \tag{6}$$

where division of the likelihood difference by n_g, the number of loci in the region g, normalises the score per locus.

In order to identify candidate alleles under selection, the sum of the non-neutrality scores from both replicas, $S_{1,g} + S_{2,g}$, was calculated for each region of the genome, ranking the results by this score, and retaining regions for which both $S_{1,g}$ and $S_{2,g}$ were greater than 0.1 (Table 2). Next, the SD model was fitted to the allele frequency data, identifying a putative locus under selection. Regions for which the driver alleles identified within both replicas were within 200kb, and for which the direction of selection was consistent between the two replicas, were retained for further investigation. On this basis, six regions of the genome were retained.

Retained regions were analysed using successively more complex models of recombination, allowing for increasing numbers of changes in the recombination rate, and performing model selection using BIC. Under this approach, the distance between

candidate alleles in the two replicas narrowed, from a mean of 87 kb to just over 17 kb (Table S1). The candidate region in chromosome IV, however, was identified as a false positive of the previous method, the SDR model suggesting selection for alleles from different parents in the two replica datasets; this region was excluded from further analysis. Increasingly complex models of recombination change were fitted to the data using BIC for model selection. Calculated BIC values are shown in Table S2, with local inferences of recombination rate given in Table S3.

Confidence intervals for allele locations

Confidence intervals for the location of each inferred selected were found by calculating likelihoods for models in which the location of the selected allele was fixed. Regions of the genome for which the calculated model likelihood was consistently within 3 log likelihood units of the maximum log likelihood were derived, corresponding roughly to a 99% confidence interval.

A first confidence interval was generated in this manner by forcing the location of the selected allele to be consistent between the two replicates, and calculating the sum of the model log likelihoods for the two replicates. Allowing for the potential effects of biological noise in the data, a second, more conservative interval was also generated, representing the span of alleles for which the likelihood calculated in either replicate was within 3 log likelihood units of the maximum; this second interval becomes large when data in either one of the two experiments is ambiguous about the allele location. Confidence intervals are illustrated in Figure 3 of the main text.

Mathematical models of allele frequency change

For convenience, we denote the 17X allele at any locus as 1, and the CU allele as 0. Thus, at a given locus i we denote the frequency of the 17X allele, as x_i^1, and the frequency of the CU allele as x_i^0. Given a set of two loci, i and j, we denote the frequency of individuals with allele a at locus i and allele b at locus j as x_{ij}^{ab}, where a and b are either 0 or 1.

We assume that, before the cross occurs, changes in the frequency of the CU and 17X malaria types may occur due to selection upon one type or another. At the time of the cross, we assume that the frequency of 17X types is equal to some value, X, where $0 \leq X \leq 1$. Following the cross, the population comprises a fraction X^2 of pure 17X individuals, $(1-X)^2$ pure CU individuals, and $2X(1-X)$ individuals which have undergone crossing. Subsequent selection can change both the fraction of pure types in the population, and the composition of the crossed individuals.

Neutral model

The neutral model assumes that a given region of the genome does not contain an allele under selection. Under this model, over the course of time, allele frequencies in the region can change, but only due to selection upon pure types acting at alleles elsewhere in the genome. In consequence, the allele frequencies are expected to remain uniform across the region. We describe the allele frequencies as

$$x_i^1 = x \ \ \forall i, \tag{7}$$

learning the value of the frequency parameter x.

Single driver model

Given a region of the genome, we suppose that the allele 1 at locus i is under selection, with strength σ (which may be positive or negative).

We denote the time of the cross as t_c. Following the cross, the selected allele is modeled as changing frequency deterministically according to the equation

$$x_i^1(t) = \frac{Xe^{\sigma(t-t_c)}}{1 - X + Xe^{\sigma(t-t_c)}} \tag{8}$$

We denote the frequency of this allele at the time of observation as $x_i^1(t_o)$.

Between t_c and t_o, the frequency of an allele $j \neq i$, while not itself under selection, will change via linkage disequilibrium with the allele at i, as described by the equation

$$x_j^1(t) = x_i^1(t)\frac{x_{ij}^{11}(t_c)}{x_i^1(t_c)} + x_i^0(t)\frac{x_{ij}^{01}(t_c)}{x_i^0(t_c)} \tag{9}$$

To calculate the haplotype frequencies, $x_{ij}^{11}(t_c)$ and $x_{ij}^{01}(t_c)$, we consider separately the pure and crossed genotypes. The pure genotypes contribute a frequency X^2 towards the frequency $x_{ij}^{11}(t_c)$, but make no contribution to the frequency $x_{ij}^{01}(t_c)$. Considering allele frequencies among the crossed fraction of the population, we denote by $\tilde{x}_i^1$ the frequency of the allele 1 at the locus i within the crossed individuals alone. Following the cross, we have that

$$\tilde{x}_{ij}^{11}(t_c) = \tilde{x}_i^1(t_c)\tilde{x}_j^1(t_c) + D'_{ij}e^{-\rho\Delta_{ij}}, \tag{10}$$

where ρ is the rate of recombination per site per generation, Δ_{ij} is the sequence length between the loci i and j, and D'_{ij} is the linkage disequilibrium between alleles at i and j before the cross. Assuming that no selection took place during the crossing procedure, we have

$$\tilde{x}_i^1(t_c) = \tilde{x}_j^1(t_c) = 0.5. \tag{11}$$

Furthermore, the mating process involves equal numbers of pure types, so that $D'_{ij} = 0.25$. We thus have the result

$$\tilde{x}_{ij}^{11}(t_c) = \frac{1}{4}(1 + e^{-\rho\Delta_{ij}}), \tag{12}$$

and, combining the cross and pure types,

$$x_{ij}^{11}(t_c) = X^2 + \frac{1}{2}X(1-X)(1 + e^{-\rho\Delta_{ij}}). \tag{13}$$

In a similar manner, we obtain the result

$$\tilde{x}_{ij}^{01}(t_c) = \tilde{x}_i^0(t_1)\tilde{x}_j^1(t_c) - D'_{ij}e^{-\rho\Delta_{ij}} = \frac{1}{4}(1 - e^{-\rho\Delta_{ij}}) \tag{14}$$

so that

$$x_{ij}^{01}(t_c) = \frac{1}{2}X(1-X)(1 - e^{-\rho\Delta_{ij}}). \tag{15}$$

Combining these terms, and remembering that $x_i^1(t_c) = X$, while $x_i^0(t_c) = 1 - X$, we derive the equation

$$x_j^1(t) = \left[X + \frac{1}{2}(1-X)(1 + e^{-\rho\Delta_{ij}})\right] x_i^1(t) + \left[\frac{1}{2}X(1 - e^{-\rho\Delta_{ij}})\right] x_i^0(t) \tag{16}$$

We add to this one other term, e, denoting the effect of selection acting upon loci in other chromosomes upon the frequencies of the pure genotypes, obtaining the final model

$$\begin{aligned} x_i^1(t_o) &= x + e && (17) \\ x_j^1(t_o) &= \left[X + \frac{1}{2}(1-X)(1 + e^{-\rho\Delta_{ij}})\right] x + \left[\frac{1}{2}X(1 - e^{-\rho\Delta_{ij}})\right](1-x) + e && (18) \end{aligned}$$

where x is equivalent to $x_i^1(t_o)$ in the model above. To specify the model, it is sufficient to learn the parameters i, X, x, ρ and e, where i denotes a locus in the given genomic region,$0 \leq X \leq 1$, $-X^2 \leq e \leq (1-X)^2$, $X^2 \leq x \leq (1-X)^2$, and $0 \leq x+e \leq 1$.

Single-driver with variable recombination rate

The models above assume that the rate of recombination during the cross is constant within each chromosome. However, where the rate of recombination is variable, such an assumption can lead to incorrect placement of the locus under selection. We therefore developed a hierarchy of SD models, allowing for variable recombination rate. In the kth such model, we learnt k recombination rates $\rho_1, \ldots, \rho_k$,and $k-1$ loci, $i_{\rho_1}, \ldots, i_{\rho_{k-1}}$, such that, where i_{ρ_0} and i_{ρ_k} are defined as the first and last loci in the genomic region, the recombination rate between locus i_{ρ_j} and $i_{\rho_{j+1}}$ was equal to ρ_{j+1}. Mathematically, such a model is identical to the SD model described above, except that the term $\rho\Delta_{ij}$, describing the breakage in linkage disequilibrium between loci i and j, is replaced by the sum

$$\sum_{k=1}^{K} \rho_{n_k} \tag{19}$$

where ρ_{n_k} is the recombination rate between the alleles n_k and n_{k+1}, $n_1 = i$ and $n_K = j$. We denote the SD model with one change of recombination rate as the SDR model, the SD model with two changes of recombination rate as the SD2R model, and so forth.

References

1. Fischer A, Vázquez-García I, Illingworth CJ, Mustonen V. High-Definition Reconstruction of Clonal Composition in Cancer. Cell Rep. 2014 Jun 12;7(5):1740–1752.

2. Illingworth CJ, Parts L, Schiffels S, Liti G, Mustonen V. Quantifying selection acting on a complex trait using allele frequency time series data. Mol Biol Evol. 2012 Apr;29(4):1187–1197.
