# Supplementary material for: Rapid identification of genes controlling virulence and immunity in malaria parasites
Source: PLoS Pathog. 2017 Jul 12;13(7):e1006447. doi: 10.1371/journal.ppat.1006447 (PMC5507557; doi:10.1371/journal.ppat.1006447)
Supplement: S1 Fig — (PDF) [file ppat.1006447.s002.pdf]

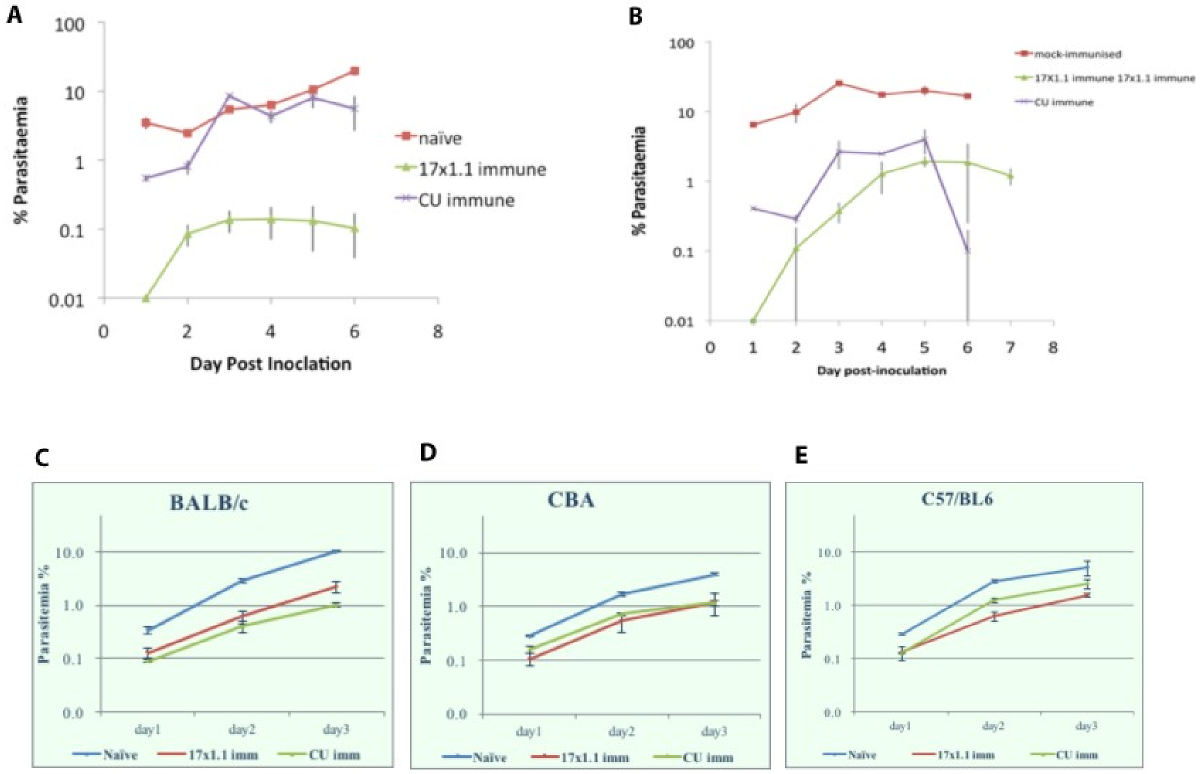

Figure S1. Parasitaemias after immune challenges. (A) The course of infection of 1:1 mixtures of blood stage *Plasmodium yoelii yoelii* 17x1.1 and CU parasites in mock-immunised (red line), 17x1.1 (green line) and CU (purple line) immunised mice through time. Error bars indicate standard errors of the mean of 6 mice per group. (**B)** The course of infection of uncloned recombinant progeny of a cross between *Plasmodium yoelii yoelii* 17x1.1 and CU parasites in mock-immunised (red line), 17x1.1 (green line) and CU (purple line) immunised mice through time. (**C-E)** The course of infection of 1:1 mixtures of blood stage *Plasmodium yoelii yoelii* 17x1.1 and CU parasites in mock-immunised (blue lines), 17x1.1 (red lines) and CU (green lines) immunised mice through time in BALB/c (**C**), CBA/n (**D**) and C57/BL6 (**E**) mice. Error bars indicate standard errors of the mean of 3 mice per group.
