# Supplementary material for: Rapid identification of genes controlling virulence and immunity in malaria parasites
Source: PLoS Pathog. 2017 Jul 12;13(7):e1006447. doi: 10.1371/journal.ppat.1006447 (PMC5507557; doi:10.1371/journal.ppat.1006447)
Supplement: S2 Fig — (PDF) [file ppat.1006447.s003.pdf]

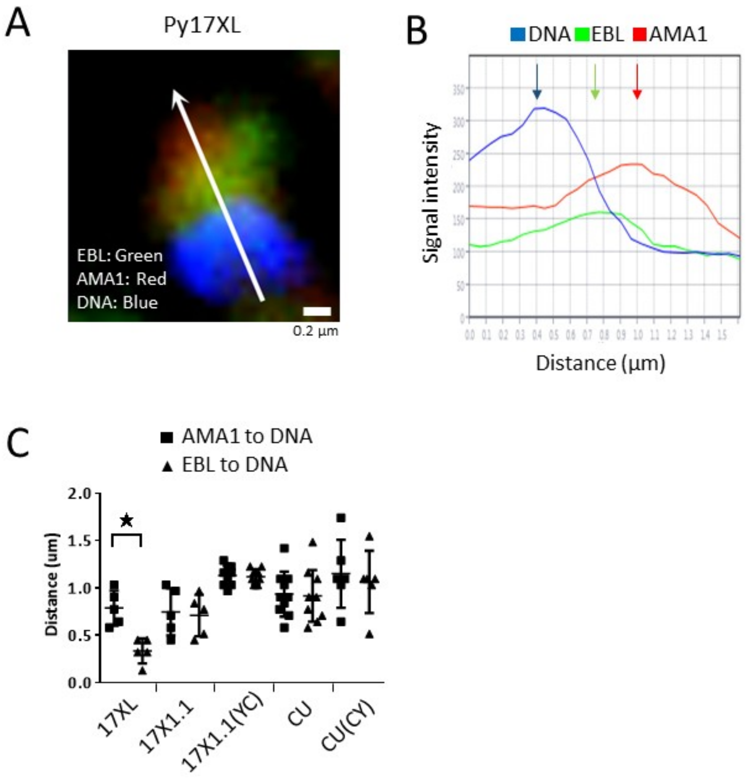

Figure S1. Intracellular localization of EBL in parasite strains CU, 17XL, 17X1.1pp and in transfected parasites CU(CY) and 17X1.1pp(YC). (A) Antibody-mediated staining of EBL (green), AMA1 (red) and DAPI staining of DNA (blue) inside the parasite cell in strain 17XL.(B) Intensity of fluorescent staining related to location in strain 17XL, Y-axis indicates fluorescence intensity, X-axis indicates distance along the merozoite starting from the posterior terminal end. (C) Comparisons of the distances of EBL from DNA and AMA1 from DNA in the 5 parasite strains. The distance of EBL or AMA1 from DNA measured across 5 parasite strains and between 5-9 merozoites for each strain; stars indicate $p<0.05$ using a Wilcoxon signed-rank test.
