# Supplementary material for: Rapid identification of genes controlling virulence and immunity in malaria parasites
Source: PLoS Pathog. 2017 Jul 12;13(7):e1006447. doi: 10.1371/journal.ppat.1006447 (PMC5507557; doi:10.1371/journal.ppat.1006447)
Supplement: S3 Fig — (PDF) [file ppat.1006447.s004.pdf]

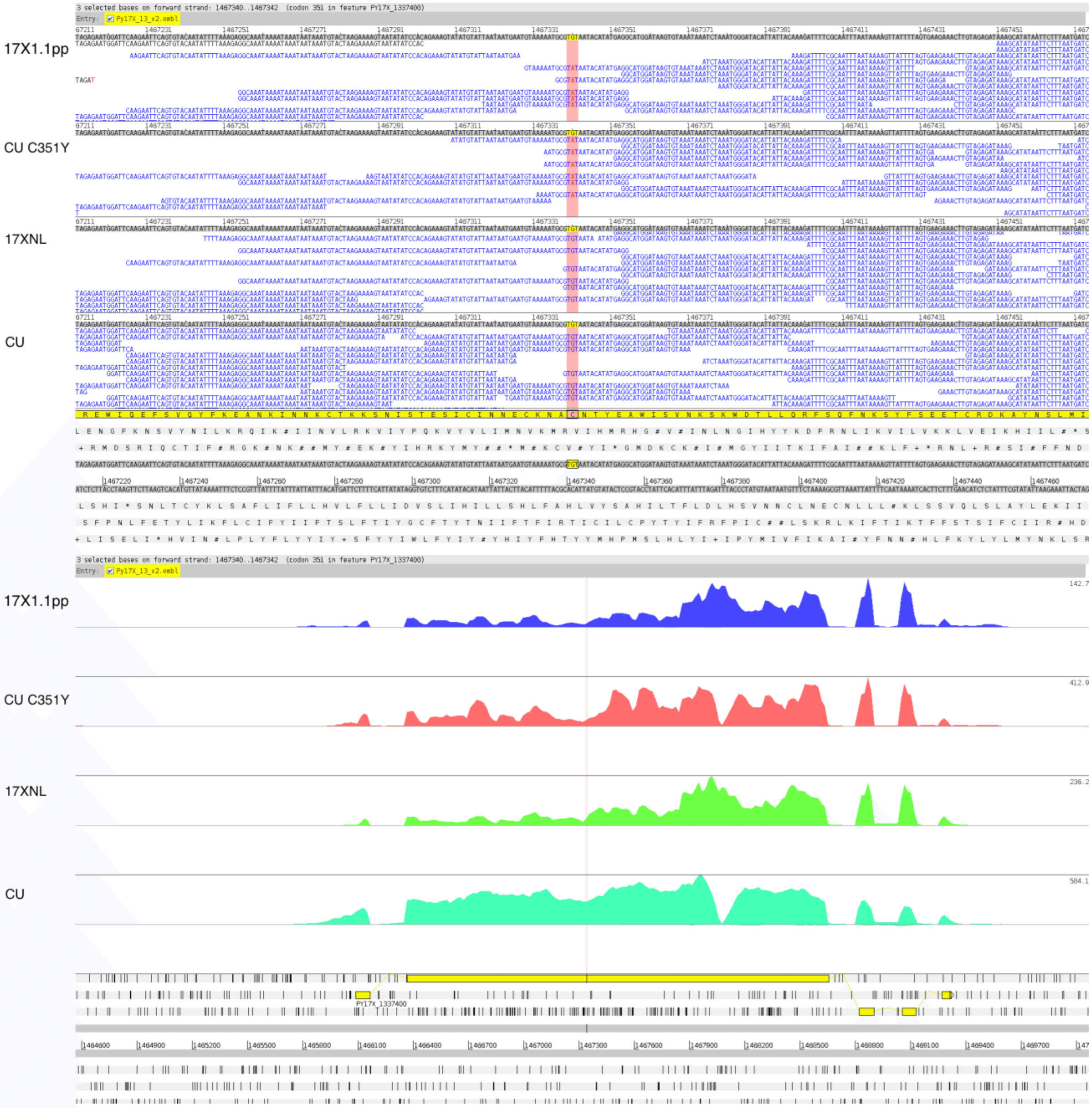

Figure S2. Expression of Pyebl alleles in both wild type (WT) and transfected strains. mRNA from the parental WT strains CU and 17X1.1pp, as well the CU strain transfected with the 17X1.1pp allele (CU C351Y) and the 17XNL strain (which also carries a C at position 351) was sequenced by strand-specific RNA sequencing. Reads were visualized on the genome using the Artemis software. (**A**) Each strain displays the expected allele at position 351 (highlighted in red) of the Pyebl gene. (**B**) The *pyebl* gene is expressed in all samples, including the transfected CU strain (CU C351Y).
