# Supplementary material for: Rapid identification of genes controlling virulence and immunity in malaria parasites
Source: PLoS Pathog. 2017 Jul 12;13(7):e1006447. doi: 10.1371/journal.ppat.1006447 (PMC5507557; doi:10.1371/journal.ppat.1006447)
Supplement: S1 Table — (PDF) [file ppat.1006447.s005.PDF]

Table S1. Selected alleles identified by the SDR model. The identified alleles are substantially closer than those identified with the more basic SD model (†indicates that the identified selected alleles were under selection for alleles from different parents).

**Condition**	**Chromosome**	**Locus** $\mathbf{i_1^*}$ (kb)	**Locus** $\mathbf{i_2^*}$ (kb)
Naïve	XIII_1	1513.234	1528.510
17X-immunised	XIII_1	1510.765	1482.441
17X-immunised	VIII_2	1289.068	1288.240
17X-immunised	VII_2	730.920	733.968
17X-immunised	IV_1	266.128	246.153†
CU-immunised	VIII	1327.639	1292.279
