# Supplementary material for: Rapid identification of genes controlling virulence and immunity in malaria parasites
Source: PLoS Pathog. 2017 Jul 12;13(7):e1006447. doi: 10.1371/journal.ppat.1006447 (PMC5507557; doi:10.1371/journal.ppat.1006447)
Supplement: S2 Table — (PDF) [file ppat.1006447.s006.PDF]

Table S2. Bayesian Information Criterion (BIC) values for varying models for candidate regions of the genome, within each replica, calculated under different models. BIC scores are given for the maximum likelihood candidate allele, i^* found within each region, in each replica. Optimal BIC scores for each genomic region within each replica, are given in bold text. In the first part of chromosome VIII, and the second part of chromosome XIII, a candidate allele could only be identified in only one of the two replicas.

**Condition**	**Chromosome**	**Allele range**	**Model M**	$\mathbf{BIC^M_{1,i^*}}$	$\mathbf{BIC^M_{2,i^*}}$
Naïve	XIII	702444 - 1721212	SD	4597.01	5688.37
Naïve	XIII	702444 - 1721212	SDR	4508.42	**5670.09**
Naïve	XIII	702444 - 1721212	SD2R	**4490.42**	5674.58
Naïve	XIII	702444 - 1721212	SD3R	4537.65	5693.42
17X-immunised	VII	571238 - 851345	SD	2588.36	3339.47
17X-immunised	VII	571238 - 851345	SDR	**2549.5**	**3289.1**
17X-immunised	VII	571238 - 851345	SD2R	2560.85	3299.4
17X-immunised	VIII	1102758 - 1523696	SD	3892.99	5536.69
17X-immunised	VIII	1102758 - 1523696	SDR	3874.91	5518.77
17X-immunised	VIII	1102758 - 1523696	SD2R	**3852.96**	**5482.06**
17X-immunised	VIII	1102758 - 1523696	SD3R	3869.74	5516.66
17X-immunised	XIII	701911 - 1721212	SD	6119.6	8542.22
17X-immunised	XIII	701911 - 1721212	SDR	5423.56	8341.43
17X-immunised	XIII	701911 - 1721212	SD2R	**5413.24**	8273.25
17X-immunised	XIII	701911 - 1721212	SD3R	5415.88	**8244.95**
CU-immunised	VIII	881993 - 1523696	SD	2900.88	**3882.93**
CU-immunised	VIII	881993 - 1523696	SDR	**2886.43**	3887.22
CU-immunised	VIII	881993 - 1523696	SD2R	2895.96	3895.65
