# Supplementary material for: Rapid identification of genes controlling virulence and immunity in malaria parasites
Source: PLoS Pathog. 2017 Jul 12;13(7):e1006447. doi: 10.1371/journal.ppat.1006447 (PMC5507557; doi:10.1371/journal.ppat.1006447)
Supplement: S3 Table — (PDF) [file ppat.1006447.s007.PDF]

Table S3. Inferred recombination rates from driver models. Recombination rates were inferred close to selected loci within each cross population. A step-wise model of recombination was applied. Recombination rates are described as number of events per base per generation.

Dataset (replica)	Chromosome	Recombination rate			
(replica)		(location kb)	(location kb)	(location kb)	(location kb)
Naïve(1)	XIII	4.673×10^{-6} (702.444 – 1419.502)	4.668×10^{-7} (1419.502 – 1633.584)	1.521×10^{-5} (1633.584 – 1721.212)	
Naïve(2)	XIII	1.878×10^{-12} (702.444 – 1266.012)	1.429×10^{-6} (1266.012 – 1721.212)		
17X-immunised(1)	VII	4.092×10^{-5} (571.238 – 744.259)	3.543×10^{-11} (744.259 – 851.345)		
17X-immunised(2)	VII	5.924×10^{-5} (571.238 – 738.215)	2.384×10^{-7} (738.215 – 851.345)		
17X-immunised(1)	VIII	1.780×10^{-5} (1102.758 – 1227.028)	5.814×10^{-7} (1227.028 – 1371.311)	3.202×10^{-5} (1371.311 – 1523.696)	
17X-immunised(2)	VIII	4.266×10^{-5} (1102.758 – 1186.367)	5.313×10^{-6} (1186.367 – 1331.769)	1.930×10^{-5} (1331.769 – 1523.696)	
17X-immunised(1)	XIII	5.250×10^{-7}) (702.444 – 1237.400)	1.057×10^{-6} (1237.400 – 1603.814)	1.254×10^{-5} (1603.814 – 1721.212)	
17X-immunised(2)	XIII	4.383×10^{-7} (702.444 – 1294.327)	4.235×10^{-6} (1294.327 – 1443.731)	1.178×10^{-6} (1443.731 – 1615.892)	2.035×10^{-5} (1615.892 – 1721.212)
CU-immunised(1)	VIII	3.633×10^{-7} (881.993 – 1043.904)	7.192×10^{-7} (1043.904– 1523.696)		
CU-immunised(2)	VIII	8.991×10^{-6} (881.993 – 1523.696)			
