# Supplementary material for: Rapid identification of genes controlling virulence and immunity in malaria parasites
Source: PLoS Pathog. 2017 Jul 12;13(7):e1006447. doi: 10.1371/journal.ppat.1006447 (PMC5507557; doi:10.1371/journal.ppat.1006447)
Supplement: S4 Table — (PDF) [file ppat.1006447.s008.pdf]

CHRVII

[Gene ID]	[Genomic Location(s)]	[Product Description]	[# TM Domains]	[Signal Peptide]	P. falciparum orthologue	NS/S SNP Ratio P.f.
PY17X_0720000	Py17X_07_v2: 724,964 - 727,213 (+)	conserved Plasmodium protein, unknown function	0	NO	PF3D7_0417900	5.06
PY17X_0720100	Py17X_07_v2: 727,812 - 742,672 (+)	conserved Plasmodium protein, unknown function	2	YES	PF3D7_0418000	2.78
PY17X_0720200	Py17X_07_v2: 743,290 - 746,481 (-)	conserved Plasmodium protein, unknown function	0	NO	PF3D7_0418100	2.93
PY17X_0720300	Py17X_07_v2: 749,073 - 750,377 (+)	eukaryotic translation initiation factor 3 subunit M, putative (EIF3M)	0	NO	PF3D7_0418200	2.14
PY17X_0720400	Py17X_07_v2: 751,266 - 753,446 (-)	conserved Plasmodium protein, unknown function	0	NO	PF3D7_0418300	2.14
PY17X_0720500	Py17X_07_v2: 759,308 - 759,900 (+)	conserved Plasmodium protein, unknown function	0	NO	PF3D7_0418400	3.5
PY17X_0720600	Py17X_07_v2: 760,071 - 761,364 (-)	trafficking protein particle complex subunit 3, putative (BET3)	0	NO	PF3D7_0418500	0.4
PY17X_0720700	Py17X_07_v2: 763,162 - 767,718 (+)	regulator of chromosome condensation, putative	0	NO	PF3D7_0418600	2.14
PY17X_0720800	Py17X_07_v2: 768,446 - 769,921 (+)	RNA-binding protein NOB1, putative	0	NO	PF3D7_0418700	4.26
PY17X_0720900	Py17X_07_v2: 770,582 - 772,423 (+)	RNA-binding protein NOB1, putative	2	YES	PF3D7_0418800	0.73
PY17X_0721000	Py17X_07_v2: 773,812 - 775,583 (+)	conserved Plasmodium protein, unknown function	0	NO	PF3D7_0418900	2.29
PY17X_0721100	Py17X_07_v2: 776,551 - 779,153 (+)	conserved Plasmodium protein, unknown function	0	NO	PF3D7_0419000	1.16
PY17X_0721200	Py17X_07_v2: 779,446 - 780,461 (+)	conserved Plasmodium protein, unknown function	0	NO	PF3D7_0419100	1.89
PY17X_0721300	Py17X_07_v2: 781,850 - 782,260 (+)	CGI-141 protein homolog, putative	3	NO	PF3D7_0419200	0
PY17X_0721400	Py17X_07_v2: 782,903 - 784,002 (-)	conserved Plasmodium protein, unknown function	0	NO	PF3D7_0419300	1.33
PY17X_0721500	Py17X_07_v2: 784,994 - 791,991 (+)	conserved Plasmodium protein, unknown function	2	YES	PF3D7_0419400	2.64
PY17X_0721600	Py17X_07_v2: 792,550 - 794,133 (-)	conserved Plasmodium protein, unknown function	5	NO	PF3D7_0419500	1.35
PY17X_0721700	Py17X_07_v2: 795,969 - 797,152 (+)	ran binding protein 1, putative	0	NO	PF3D7_0419600	2.31
PY17X_0721800	Py17X_07_v2: 799,281 - 800,081 (+)	apical merozoite protein, putative	2	YES	PF3D7_0419700	1.93
PY17X_0721900	Py17X_07_v2: 801,271 - 801,981 (-)	60S ribosomal protein L7ae/L30e, putative	0	NO	PF3D7_0419800	1.83
PY17X_0722000	Py17X_07_v2: 804,041 - 818,638 (+)	phosphatidylinositol 4-kinase, putative	18	NO	PF3D7_0419900	3.49

Table S4. List of genes contained within the mathematically defined Confidence Intervals (725,528-813,866 bp) of the locus under selection on Chromosome 7.
The table shows gene ID and location for *P. yoelii*, protein description, number of Transmembrane domains, presence of a signal peptide, *P. falciparum* orthologous gene and non-synonymous to synonymous SNP ratio in *P. falciparum*
