# Supplementary material for: Rapid identification of genes controlling virulence and immunity in malaria parasites
Source: PLoS Pathog. 2017 Jul 12;13(7):e1006447. doi: 10.1371/journal.ppat.1006447 (PMC5507557; doi:10.1371/journal.ppat.1006447)
Supplement: S5 Table — (PDF) [file ppat.1006447.s009.pdf]

CHRVIII

[Gene ID]	[Genomic Location(s)]	[Product Description]	[# TM Domains]	[Signal Peptide]	P. falciparum orthologue	NS/S SNP Ratio P.f.
PY17X_0832800	Py17X_08_v2: 1,228,623 - 1,232,366 (-)	conserved Plasmodium protein, unknown function	2	NO	PF3D7_0928700	3.23
PY17X_0832900	Py17X_08_v2: 1,233,777 - 1,235,606 (+)	serine/threonine protein kinase, putative	0	NO	PF3D7_0928800	3.54
PY17X_0833000	Py17X_08_v2: 1,236,610 - 1,237,206 (+)	guanylate kinase, putative (GK)	0	NO	PF3D7_0928900	0.45
PY17X_0833100	Py17X_08_v2: 1,237,965 - 1,239,388 (+)	transcription initiation factor TFIID subunit 7, putative (TAF7)	0	NO	PF3D7_0929000	2
PY17X_0833200	Py17X_08_v2: 1,240,649 - 1,242,055 (+)	conserved Plasmodium protein, unknown function	0	NO	PF3D7_0929100	0.92
PY17X_0833300	Py17X_08_v2: 1,243,623 - 1,245,309 (+)	RNA-binding protein, putative	0	NO	PF3D7_0929200	1.63
PY17X_0833400	Py17X_08_v2: 1,246,772 - 1,248,454 (+)	conserved Plasmodium protein, unknown function	0	NO	PF3D7_0929300	4.38
PY17X_0833500	Py17X_08_v2: 1,249,994 - 1,255,231 (+)	high molecular weight rhoptry protein 2 (RhopH2)	0	YES	PF3D7_0929400	1.6
PY17X_0833600	Py17X_08_v2: 1,255,518 - 1,256,536 (-)	conserved protein, unknown function	0	NO	PF3D7_0929500	1.4
PY17X_0833700	Py17X_08_v2: 1,257,624 - 1,258,566 (-)	G2 protein, putative	0	NO	PF3D7_0929600	0.75
PY17X_0833800	Py17X_08_v2: 1,260,208 - 1,261,242 (-)	conserved Plasmodium protein, unknown function	0	NO	PF3D7_0929700	2.86
PY17X_0833900	Py17X_08_v2: 1,261,874 - 1,262,170 (-)	conserved Plasmodium protein, unknown function	1	NO	PF3D7_0929800	0
PY17X_0834000	Py17X_08_v2: 1,262,968 - 1,263,982 (+)	conserved Plasmodium protein, unknown function, fragment	2	YES	PF3D7_0929900	3
PY17X_0834100	Py17X_08_v2: 1,264,568 - 1,266,140 (+)	procollagen lysine 5-dioxygenase, putative	2	NO	PF3D7_0930000	1.71
PY17X_0834200	Py17X_08_v2: 1,266,238 - 1,269,543 (-)	conserved Plasmodium protein, unknown function	0	YES	PF3D7_0930100	2.52
PY17X_0834300	Py17X_08_v2: 1,270,149 - 1,272,992 (-)	leucine-rich repeat protein (LRR8)	0	NO	PF3D7_0930200	3.26
PY17X_0834400	Py17X_08_v2: 1,276,004 - 1,281,322 (+)	merozoite surface protein 1 (MSP1)	1	YES	PF3D7_0930300	8.43
PY17X_0834500	Py17X_08_v2: 1,282,398 - 1,285,597 (+)	conserved Plasmodium protein, unknown function	0	NO	PF3D7_0930400	3.51
PY17X_0834600	Py17X_08_v2: 1,286,002 - 1,289,987 (-)	diacylglycerol kinase, putative	2	NO	PF3D7_0930500	2.12
PY17X_0834700	Py17X_08_v2: 1,291,496 - 1,293,856 (-)	peptidyl-prolyl cis-trans isomerase, putative (CYP72)	0	NO	PF3D7_0930600	4.13
PY17X_0834800	Py17X_08_v2: 1,294,482 - 1,296,023 (+)	conserved Plasmodium protein, unknown function	0	NO	PF3D7_0930700	1.92
PY17X_0834900	Py17X_08_v2: 1,297,526 - 1,310,388 (+)	conserved Plasmodium protein, unknown function	8	NO	PF3D7_0930800	2.4
PY17X_0835000	Py17X_08_v2: 1,315,116 - 1,316,378 (-)	conserved Plasmodium protein, unknown function	0	NO	NONE	NA
PY17X_0835100	Py17X_08_v2: 1,317,930 - 1,318,619 (-)	NifU-like protein, putative	0	NO	PF3D7_0930900	2.2
PY17X_0835200	Py17X_08_v2: 1,319,633 - 1,322,074 (-)	elongation factor Tu, putative	0	NO	PF3D7_0931000	3.6
PY17X_0835300	Py17X_08_v2: 1,323,871 - 1,325,769 (+)	nucleolar protein Nop52, putative	0	NO	PF3D7_0931100	5.24
PY17X_0835400	Py17X_08_v2: 1,326,314 - 1,327,072 (+)	selenoprotein, putative (Sel2)	3	YES	PF3D7_0931200	1.8
PY17X_0835500	Py17X_08_v2: 1,328,449 - 1,329,600 (+)	conserved Plasmodium protein, unknown function	0	YES	PF3D7_0931300	2
PY17X_0835600	Py17X_08_v2: 1,331,232 - 1,332,723 (+)	conserved Plasmodium protein, unknown function	0	NO	PF3D7_0931400	1.67
PY17X_0835700	Py17X_08_v2: 1,333,120 - 1,334,106 (-)	conserved Plasmodium protein, unknown function	2	YES	PF3D7_0931500	0.57
PY17X_0835800	Py17X_08_v2: 1,336,199 - 1,337,061 (+)	conserved protein, unknown function	0	NO	PF3D7_0931600	3
PY17X_0835900	Py17X_08_v2: 1,337,701 - 1,339,975 (+)	PIH1 domain-containing protein, putative	0	NO	PF3D7_0931700	1.93
PY17X_0836000	Py17X_08_v2: 1,340,434 - 1,341,261 (-)	proteasome subunit beta type-6, putative	0	NO	PF3D7_0931800	0.71
PY17X_0836100	Py17X_08_v2: 1,342,693 - 1,343,945 (-)	adenylate kinase-like protein 2, putative (AKLP2)	0	NO	PF3D7_0931900	1.15
PY17X_0836200	Py17X_08_v2: 1,345,851 - 1,347,009 (+)	conserved Plasmodium protein, unknown function	1	NO	PF3D7_0932000	2.33
PY17X_0836300	Py17X_08_v2: 1,347,152 - 1,350,626 (-)	protein MAM3, putative	4	YES	PF3D7_0932100	2.34
PY17X_0836400	Py17X_08_v2: 1,353,299 - 1,354,693 (+)	profilin, putative (PFN)	0	NO	PF3D7_0932200	1
PY17X_0836500	Py17X_08_v2: 1,355,520 - 1,357,025 (-)	M18 aspartyl aminopeptidase, putative (M18AAP)	0	NO	PF3D7_0932300	0.71
PY17X_0836600	Py17X_08_v2: 1,358,362 - 1,359,757 (-)	peptide release factor, putative	0	NO	PF3D7_0932400	1.75
PY17X_0836700	Py17X_08_v2: 1,360,000 - 1,362,266 (-)	palmitoyltransferase, putative (DHHC6)	5	YES	PF3D7_0932500	1.84
PY17X_0836800	Py17X_08_v2: 1,363,086 - 1,363,927 (-)	apicoplast ribosomal protein S6, putative (RPS6)	1	YES	PF3D7_0932600	1.33

Table S5. List of genes contained within the mathematically defined Confidence Intervals (1,229,582 –1,363,920 bp) of the locus under selection on Chromosome 8.
The table shows gene ID and location for *P. yoelii*, protein description, number of Transmembrane domains, presence of a signal peptide, *P. falciparum* orthologous gene and non-synonymous to synonymous SNP ratio in *P. falciparum*
