# Supplementary material for: Rapid identification of genes controlling virulence and immunity in malaria parasites
Source: PLoS Pathog. 2017 Jul 12;13(7):e1006447. doi: 10.1371/journal.ppat.1006447 (PMC5507557; doi:10.1371/journal.ppat.1006447)
Supplement: S6 Table — (PDF) [file ppat.1006447.s010.pdf]

CHRXIII

[Gene ID]	[Genomic Location(s)]	[Product Description]	[# TM Domains]	[Signal Peptide]	P. falciparum orthologue	NS/S SNP Ratio P.f.
PY17X_1336500	Py17X_13_v2: 1,436,484 - 1,437,920 (-)	derlin-1, putative (DER1-1)	5	YES	PF3D7_1468500	0.67
PY17X_1336600	Py17X_13_v2: 1,443,231 - 1,444,793 (+)	helicase 45, putative (eIF4A)	0	NO	PF3D7_1468600	3.74
PY17X_1336700	Py17X_13_v2: 1,446,128 - 1,448,272 (-)	splicing factor U2AF large subunit, putative (U2AF2)	0	NO	PF3D7_1468800	1.9
PY17X_1336800	Py17X_13_v2: 1,449,742 - 1,452,081 (-)	conserved Plasmodium protein, unknown function	0	NO	PF3D7_1468900	2.26
PY17X_1336900	Py17X_13_v2: 1,454,106 - 1,454,600 (-)	translation initiation factor IF-1, putative (IF1)	1	YES	PF3D7_1469000	3
PY17X_1337000	Py17X_13_v2: 1,455,848 - 1,456,606 (+)	conserved Plasmodium protein, unknown function	1	NO	PF3D7_1469100	1.67
PY17X_1337100	Py17X_13_v2: 1,457,455 - 1,458,543 (-)	shewanella-like protein phosphatase 1, putative (SHLP1)	0	YES	PF3D7_1469200	1.13
PY17X_1337200	Py17X_13_v2: 1,459,861 - 1,460,625 (-)	small subunit rRNA processing KH domain protein, putative	0	NO	PF3D7_1469300	1.27
PY17X_1337300	Py17X_13_v2: 1,462,056 - 1,464,161 (-)	conserved Plasmodium protein, unknown function	0	NO	PF3D7_1469500	5.29
PY17X_1337400	Py17X_13_v2: 1,466,087 - 1,469,314 (+)	duffy-binding protein	1	YES	NONE	NA
PY17X_1337500	Py17X_13_v2: 1,471,895 - 1,481,288 (+)	biotin carboxylase subunit of acetyl CoA carboxylase, putative (ACC)	0	YES	PF3D7_1469600	3.6
PY17X_1337600	Py17X_13_v2: 1,482,848 - 1,483,465 (+)	mediator of RNA polymerase II transcription subunit 6, putative	0	NO	PF3D7_1469700	2.83
PY17X_1337700	Py17X_13_v2: 1,483,690 - 1,484,728 (-)	conserved Plasmodium protein, unknown function	0	NO	PF3D7_1469800	2.36
PY17X_1337800	Py17X_13_v2: 1,484,814 - 1,486,343 (+)	conserved Plasmodium protein, unknown function	0	NO	PF3D7_1469900	4.56
PY17X_1337900	Py17X_13_v2: 1,486,631 - 1,487,484 (-)	autophagy-related protein 12, putative (ATG12)	0	NO	PF3D7_1470000	0
PY17X_1338000	Py17X_13_v2: 1,488,220 - 1,494,902 (+)	conserved Plasmodium protein, unknown function	2	NO	PF3D7_1470100	2.18
PY17X_1338100	Py17X_13_v2: 1,494,881 - 1,495,738 (-)	conserved Plasmodium protein, unknown function	0	NO	PF3D7_1470200	6.67
PY17X_1338200	Py17X_13_v2: 1,496,691 - 1,497,032 (+)	conserved Plasmodium protein, unknown function	0	NO	PF3D7_1470300	4.33
PY17X_1338300	Py17X_13_v2: 1,497,582 - 1,498,331 (-)	mitochondrial pyruvate carrier protein 2, putative (MPC2)	1	NO	PF3D7_1470400	0.67
PY17X_1338400	Py17X_13_v2: 1,500,315 - 1,503,383 (+)	phosphodiesterase delta (PDEdelta)	6	NO	PF3D7_1470500	1.41
PY17X_1338500	Py17X_13_v2: 1,504,752 - 1,505,941 (-)	RAP protein, putative	0	NO	PF3D7_1470600	5.57
PY17X_1338600	Py17X_13_v2: 1,507,011 - 1,508,765 (+)	conserved Plasmodium protein, unknown function	0	NO	PF3D7_1477600	2.53
PY17X_1338700	Py17X_13_v2: 1,509,206 - 1,512,689 (-)	conserved Plasmodium protein, unknown function	0	YES	PF3D7_1470800	1.18
PY17X_1338800	Py17X_13_v2: 1,514,819 - 1,515,658 (+)	proteasome subunit beta type-2, putative	0	NO	PF3D7_1470900	0.29
PY17X_1338900	Py17X_13_v2: 1,517,285 - 1,518,946 (+)	RNA 3'-terminal phosphate cyclase-like protein, putative	0	NO	PF3D7_1471000	1.38
PY17X_1339000	Py17X_13_v2: 1,520,378 - 1,521,426 (-)	exported protein 2, putative (EXP2)	0	YES	PF3D7_1471100	2.08
PY17X_1339100	Py17X_13_v2: 1,524,724 - 1,527,216 (+)	inorganic anion antiporter, putative	11	NO	PF3D7_1471200	0.76
PY17X_1339200	Py17X_13_v2: 1,527,554 - 1,529,680 (-)	conserved Plasmodium protein, unknown function	0	NO	PF3D7_1471300	4.04

Table S6. List of genes contained within the mathematically defined Confidence Intervals (1,436,717-1,528,275 bp) of the locus under selection on Chromosome 13.
The table shows gene ID and location for *P. yoelii*, protein description, number of Transmembrane domains, presence of a signal peptide, *P. falciparum* orthologous gene and non-synonymous to synonymous SNP ratio in *P. falciparum*
