# Supplementary material for: Rapid identification of genes controlling virulence and immunity in malaria parasites
Source: PLoS Pathog. 2017 Jul 12;13(7):e1006447. doi: 10.1371/journal.ppat.1006447 (PMC5507557; doi:10.1371/journal.ppat.1006447)
Supplement: S7 Table — (PDF) [file ppat.1006447.s011.PDF]

Table S7. PCR primers used to generate constructs for transfection experiments.

**Primers for generating Gateway fragments**	**Primer sequence**
B1F	ggggACAAGTTTGTACAAAAAAGCAGGCTAGGCCTAGGGCCCCATATGAATCTTtCCAATCTTTCCC
B2R	ggggACCACTTTGTACAAGAAAGCTGGGTTTAATAAAAATCTACAGGTATATATTC
B4F	ggggACAACTTTGTATAGAAAAGTTGcaaaatattgaattgaagccttatg
B1R	ggggACTGCTTTTTTGTACAAACTTGGGCCCTAGGCCTaaatatgactatgcataccatcatg
M13R.F3F	gcGAAGTTCCTATTCTtcAaAtAGTATAGGAACTTC-AGGAAACAGCTATGAC
PbDT3U.F3R	gcGAAGTTCCTATTCTtcAaAtAGTATAGGAACTTC-CCTGAAGAAGAAAAGTCCG
**Primers for site-directed mutagenesis**	**Primer sequence**
P1.F	GTATAATACATATGAGGCATGG
P1.R	GCATTTTTACATTCATTATTAATAC
P2.F	GTGTAATACATATGAGGCATGG
P2.R	GCATTTTTACATTCATTATTAATAC
**Primers for EBL sequencing**	**Primer sequence**
P3	CGATAGTGAAAATGCCAATAAATTGGG
P4	GATATGATTAATAGTTTTGGAG
P5	GAAGAAACTTGTAGAGATAAAGC
P6	CAACAGGAGGTATAATATTTG
P7	AACCGATGATAATGCTAAAAGG
P8	TTTGTTTAAAGAAGATGACGATG
P9	TCAACCTCAACCTCAATTTCCAATTC
P10	TGAATCTTTCCAATCTTTCCC
P11	CCATGTCTCTCCGTTTTCAATG
P12	CCAAATGCATAGAGTTTTATATTTTATTA
P13	TACAATTAAAATGCTACACA
P14	CCGATGGACGCACAAATGAATATG
P15	CGTTGGCTAGCATTATTATATTTT
P16	CATTATCACAAATCCAAGCATG
P17	GTTGAAGTTGGATTGATAGTTACAG
P18	CTTCATATGCTTCTTTAAATTCGATC
P19	CAATATGTTCATGATTTTTCCACCAAG
